# ExposomeMap-PM: fine and ultrafine particle-induced toxicity - from adverse outcome pathways to systems biomedicine disease models

**DOI:** 10.3389/ftox.2026.1951777

**Published:** 2026-10-27

**Authors:** Alexander Mazein, Corrado Ameli, Salla Akerblom, Claudia Gutierrez-Ortiz, Maryna Chepeleva, Benoit J. Kunath, Lu Zhang, Bharath Anila Bhuvanendran Nair, Amalie Schufri Klinkby, Thuy Thi Lai, Ahmed Abdelmonem Hemedan, Marek Ostaszewski, Marin Kuntic, Thomas Münzel, Jos Lelieveld, Katrin B. M. Frauenknecht, Adelina Rogowska-Wrzesinska, Katja M. Kanninen, Vladimir Despotovic, Reka Toth, Andreas Daiber, Petr V. Nazarov

**Affiliations:** 1 Department of Cancer Research, Luxembourg Institute of Health, Strassen, Luxembourg; 2 Department of Precision Health, Luxembourg Institute of Health, Strassen, Luxembourg; 3 Faculty of Science, Technology and Medicine, University of Luxembourg, Esch-sur-Alzette, Luxembourg; 4 Department of Medical Informatics, Luxembourg Institute of Health, Strassen, Luxembourg; 5 Department of Biochemistry and Molecular Biology, University of Southern Denmark, Odense, Denmark; 6 A.I. Virtanen Institute for Molecular Sciences, University of Eastern Finland, Kuopio, Finland; 7 Department of Transversal Translational Medicine, Luxembourg Institute of Health, Strassen, Luxembourg; 8 Luxembourg Centre for Systems Biomedicine, University of Luxembourg, Belvaux, Luxembourg; 9 ELIXIR Luxembourg, Belvaux, Luxembourg; 10 Department for Cardiology, Molecular Cardiology, University Medical Center Mainz, Mainz, Germany; 11 German Center for Cardiovascular Research (DZHK), Partner Site Rhine-Main, Mainz, Germany; 12 Atmospheric Chemistry Department, Max Planck Institute for Chemistry, Mainz, Germany; 13 Environmental Prediction Department, Cyprus Institute, Nicosia, Cyprus; 14 Institute of Neuropathology, University Medical Center of the Johannes Gutenberg University Mainz, Mainz, Germany

**Keywords:** adverse outcome pathway, air pollution, ambient particles, AOP, disease maps, disease mechanisms, fine particles, particulate matter

## Abstract

**Background:**

Air pollution is one of the leading global environmental health risks, associated with millions of premature deaths annually. It represents a substantial share of the global disease burden. Despite well-established epidemiological associations, the underlying molecular mechanisms remain only partially understood. There is a need for systematic approaches toward mechanistic understanding that connect particulate matter exposure to downstream pathways and pathophysiological effects.

**Results:**

We present ExposomeMap-PM (https://disease-maps.io/markopolo), a model of mechanistic progression from fine and ultrafine particle exposure. ExposomeMap-PM includes 304 entities and 497 processes across five mechanistic blocks. The model identifies key components, including alveolar deposition, oxidative stress, barrier disruption, mitochondrial dysfunction, and pro-inflammatory signalling. Adverse outcomes are associated with conditions such as asthma, chronic obstructive pulmonary disease, atherosclerosis and neurodegeneration. Differential-expression values from transcriptomics and proteomics datasets were overlaid onto protein, mRNA and gene nodes of ExposomeMap-PM, providing a pathway-level view of particle-induced effects. In this application, based on a single reference particle type and a short 3-day exposure, the map nodes captured a significantly enriched share of the differentially expressed transcripts: 1.50-fold over size-matched random gene sets, permutation p = 0.0074. The proteomics overlay pointed in the same direction, 1.13-fold. This indicates that the transcriptomic response is concentrated within the mechanistic scope of the map.

**Methods:**

The approach combines the adverse outcome pathway concept from toxicology with the disease map concept from systems biomedicine. The model was constructed in CellDesigner using Systems Biology Graphical Notation standard and made available online *via* the MINERVA Platform to facilitate exploration and reuse.

**Conclusion:**

The ExposomeMap-PM provides a mechanistic layer of understanding, a missing step between epidemiological evidence of particle-induced harmful effects and the molecular events that drive it and connect to outcomes. This reusable and extendable resource bridges toxicological and biomedical perspectives, advancing the understanding of how fine and ultrafine particles contribute to the onset and progression of complex diseases. It supports community annotation and is accessible online for browsing, community feedback and data visualisation.

## Introduction

Air pollution remains one of the leading global environmental health risks, associated with millions of premature deaths annually (Fuller et al., 2022) and a substantial share of disease burden expressed in disability-adjusted life years (DALYs) across respiratory, cardiovascular, and neurological conditions (GBD 2023 Disease and Injury and Risk Factor Collaborators, 2025; McDuffie et al., 2021; Sagheer et al., 2024). Despite these well-established epidemiological associations, the underlying molecular mechanisms remain only partially understood.

About 7.9 million deaths and 232 million DALYs were attributed to air pollution in 2023 (GBD 2023 Disease and Injury and Risk Factor Collaborators, 2025; Health Effects Institute, 2025). It is ranked as the leading environmental risk factor for all-cause mortality worldwide and the second largest modifiable risk factor for the total disease burden after high blood pressure (GBD 2023 Disease and Injury and Risk Factor Collaborators, 2025; Health Effects Institute, 2025). Air pollution includes various types of particles, notably combustion-derived, but also from natural and agricultural sources. The excess mortality estimates have been derived from epidemiological associations that reflect chronic exposure over many years. They are connected to persistent low-grade inflammation and oxidative stress that progressively contribute to cardiovascular diseases, respiratory diseases and cancer. Long-term exposure to ambient fine particulate matter (PM2.5) alone is linked to an estimated 4.9 million of these disease-specific deaths (Health Effects Institute, 2025). An additional one million deaths per year is linked to short-term exposure (Yu et al., 2024). Despite a slight global decline in ambient PM2.5 concentrations since 2013, attributable mortality increased by 24% over the same period, driven by population aging and growth in highly polluted regions (Health Effects Institute, 2025). These trends highlight the need for systematic approaches that connect particulate matter exposure to downstream disease pathways and for moving beyond epidemiological associations toward mechanistic understanding.

Fine and ultrafine particles, defined as particulate matter (PM) with diameters less than 2.5 µm (PM2.5) and 0.1 µm (UFP), respectively, can be present in both ambient and indoor environments. PM2.5 primarily originates from combustion processes such as coal and biomass burning, vehicle exhaust, agricultural and industrial emissions, as well as from mechanical sources like construction dust and tire wear, and from natural sources such as desert dust, volcanic emissions and sea spray. These particles partially form secondarily through atmospheric reactions involving sulfur dioxide, nitrogen oxides, ammonia and volatile organic compounds, leading to sulfates, nitrates, and secondary organic aerosols. UFPs are likewise emitted directly from combustion, especially from gasoline and diesel engines, and also arise from nucleation of gaseous precursors followed by condensational growth.

PM2.5 and UFPs affect the respiratory, cardiovascular, and neurological systems through distinct but overlapping mechanisms. In the respiratory system, both pollutants cause inflammation and oxidative stress; however UFPs penetrate deeper into the alveoli and may translocate into circulation (Fortoul et al., 2011). In the cardiovascular system, PM2.5 primarily acts through systemic inflammation triggered by lung exposure, contributing to atherosclerosis and long-term risks such as myocardial infarction. At the same time, UFPs can enter the bloodstream directly and produce acute effects such as endothelial dysfunction and autonomic imbalance (Lederer et al., 2021). PM2.5 has only limited direct access to the central nervous system (CNS). It primarily causes indirect effects in the CNS through vascular and inflammatory pathways in brain endothelium. Furthermore, UFPs can cross the blood-brain barrier or enter the brain *via* the olfactory nerve, directly inducing neuroinflammation, microglial activation, and potentially contributing to neurodegenerative diseases (Nicholson et al., 2022; Oberdörster et al., 2005).

We offer a systems biomedicine and systems toxicology resource: the ExposomeMap-PM (https://disease-maps.io/markopolo), developed within MARKOPOLO (https://markersofpollution-markopolo.eu), a Horizon Europe project focused on respiratory, cardiovascular and neurotoxicity with direct relevance to major cardiovascular outcomes, including ischemic heart disease, stroke, chronic obstructive pulmonary disease, asthma, lung cancer, and neurodegenerative disorders such as Alzheimer’s and Parkinson’s disease. MARKOPOLO investigates health effects of air pollution and traffic noise, with a focus on fine and ultrafine particulate matter, combining experimental, epidemiological and computational approaches. By systematically organizing mechanistic knowledge of PM2.5 and UFP toxicity, the ExposomeMap-PM aims to provide the missing mechanistic layer that complements population-level evidence and supports translation between toxicological, biomedical, and public health domains.

## Methods

The methodology follows the disease maps community guidelines (Mazein et al., 2023), the AOP guidelines and best practices (OECD, 2018), and is built on experience from the previous systems toxicology maps (Mazein et al., 2025, 2024). Figure 1 outlines the steps of the framework used for the ExposomeMap-PM development.

**FIGURE 1:**
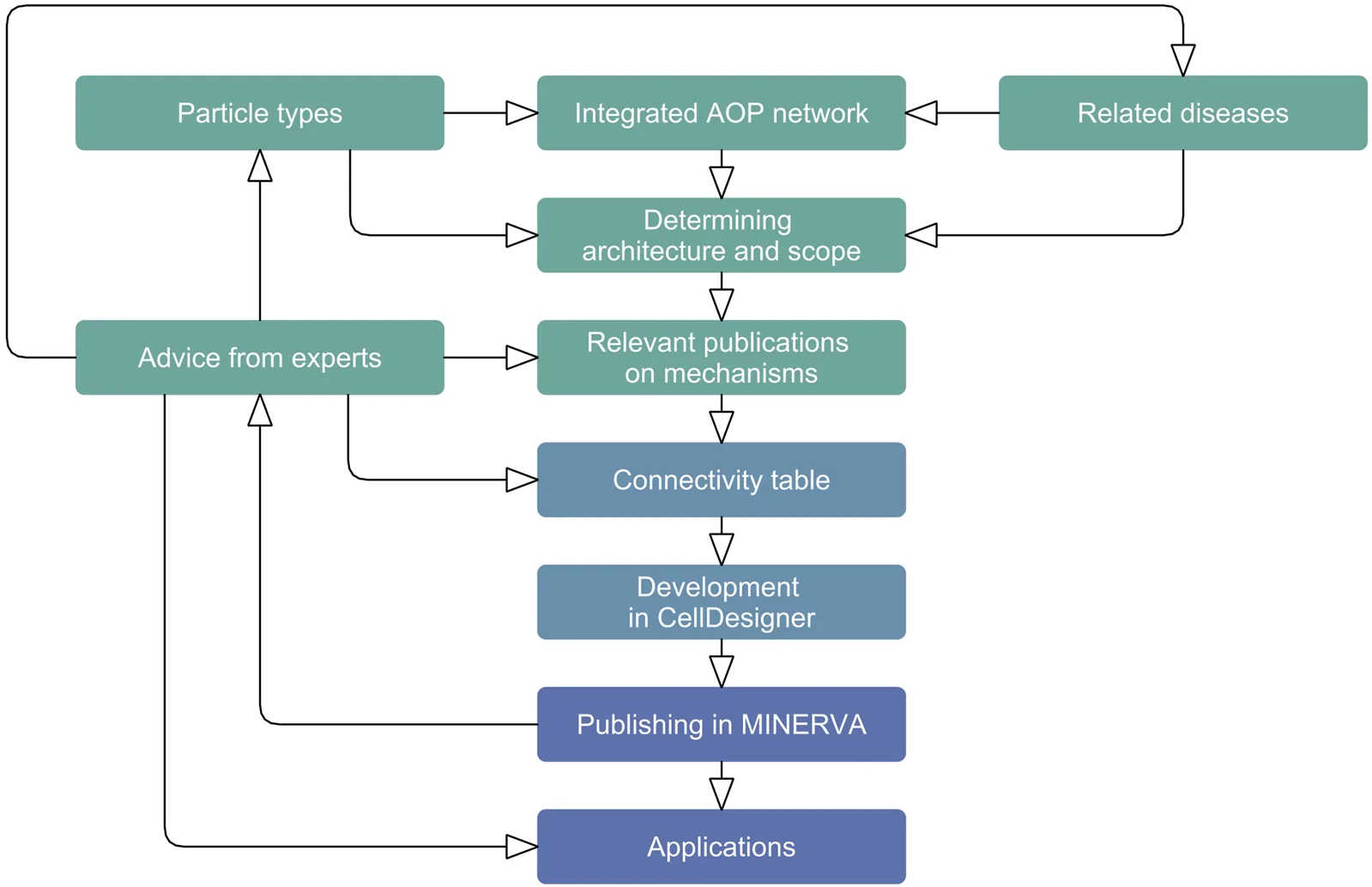
The methodological framework used for the ExposomeMap development. Detailed description of the steps are provided in the text.

The literature search followed a scoping-review strategy, consistent with the disease-map community guidelines (Mazein et al., 2023) and previous AOP-based maps (Mazein et al., 2025, 2024). The process was expert-guided and iterative: broad PubMed queries were progressively narrowed as relevant particles, receptors, mechanisms and outcomes were identified, and leads from the retrieved reviews and their cited primary articles were followed to close gaps in mechanistic continuity of the map. The search followed a defined sequence: characterising particle types, sources and chemical composition; linking particle types to receptors or receptor-independent mechanisms; identifying the relevant respiratory, cardiovascular and neurological systems phenotypic changes and associated diseases; and researching the intermediate processes connecting initiating events to the outcomes. Sources were selected for their relevance to particle-induced mechanisms across the respiratory, cardiovascular and neurological systems, with recent reviews used as entry points and the primary research they cited used for mechanism-level detail, so that mechanistic information was linked to the primary experimental works underlying the reviews. Emphasis was on literature from the past 10 years, most published between 2019 and 2025. Mechanistic studies were included irrespective of publication date.

### Integrated AOP network

The integrated AOP network (Figure 2) was created to provide a unified and harmonised representation of how molecular initiating events (MIEs) lead to key events (KEs), and ultimately, adverse health outcomes (AOs) across multiple biological systems. Existing pathway diagrams and literature-based models related to respiratory, cardiovascular, and neurologic system dysfunction were collected and reviewed as the first step for building an integrated disease map for these three systems. Components from published AOPs were reviewed (Li T et al., 2022) and merged in the freely available yEd Graph Editor (https://www.yworks.com/products/yed). Examples of the merged elements: “KE1262 Apoptosis” and “KE1918 Myocardial apoptosis” were incorporated into “KE1262 Apoptosis” in the integrated version; and “KE1927 Vascular Endothelial Cell Autophagy” and “KE1945 Dysfunctional autophagy” were merged into “KE1945 Autophagy dysfunction”. The complete list of merged, absorbed and generalised elements is provided in Supplementary Material S1, mapping each source key event to its integrated node. Gaps in the mechanistic continuity between some events were addressed by performing an additional literature search using PubMed (www.pubmed.gov) to identify plausible intermediate key events (Ding et al., 2024; Halappanavar et al., 2020; Li T et al., 2022; Yu et al., 2024; Zhang et al., 2024). This integrated AOP network provides a comprehensive visual framework linking molecular, cellular, and organ-level processes across respiratory, cardiovascular, and neurologic systems and serves as the basis for developing the ExposomeMap-PM resource.

**FIGURE 2:**
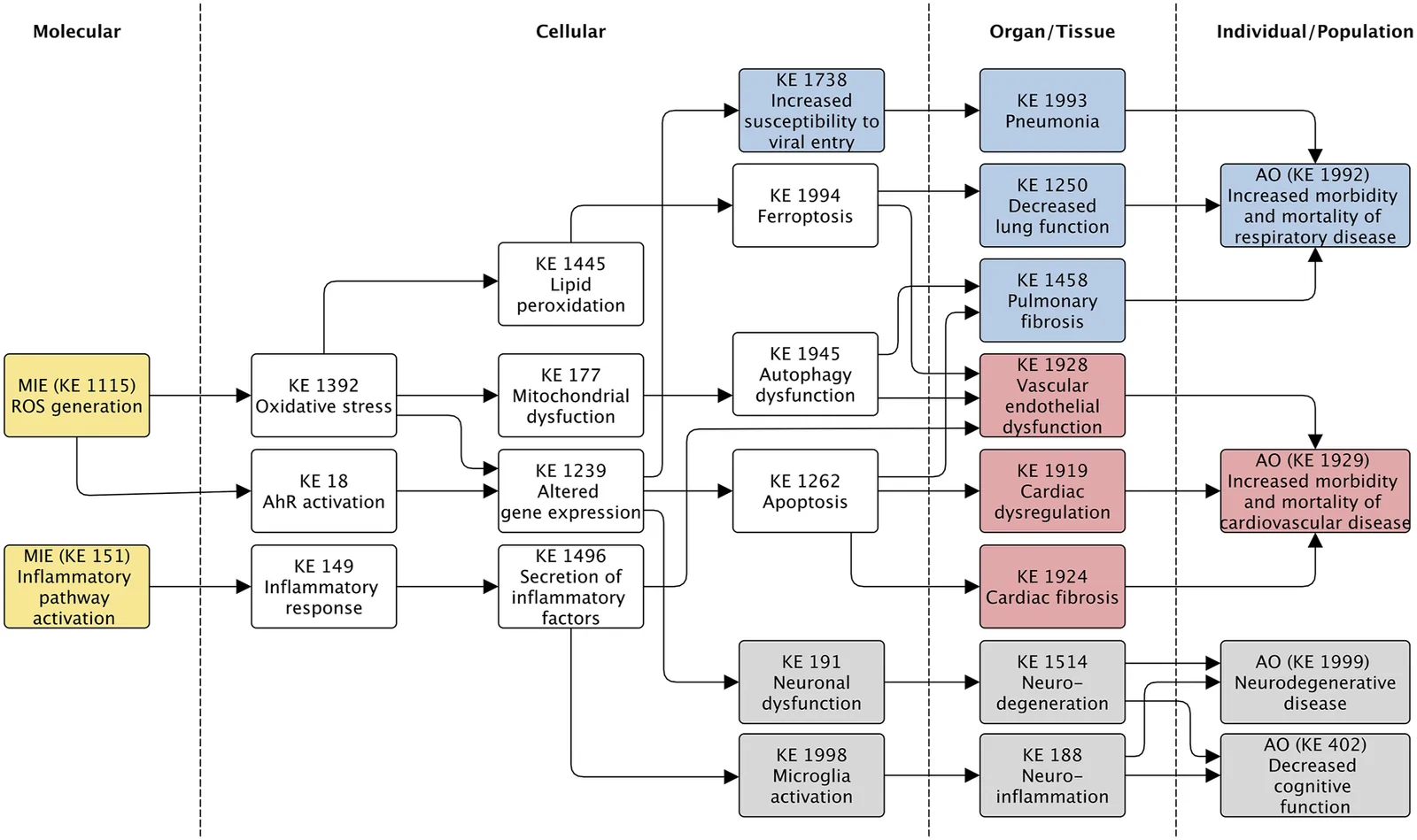
Integrated particle-matter-induced AOP network developed based on the previously published AOPs (references are provided in the text). AO, adverse outcome; MIE, molecular initiating event; KE, key event; ROS, reactive oxygen species. Codes shown on nodes (e.g., KE1115) are AOP-Wiki (https://aopwiki.org) key event identifiers from the source AOPs (Li T et al., 2022). Colour code indicates system context: yellow - initiating-event layer; blue - respiratory outcomes; red - cardiovascular outcomes; grey - neurological outcomes; white - shared cellular key events/convergence mechanisms.

### Particle types

While particle types used in the MARKOPOLO study are narrowed to pure carbon and silica (https://tsapps.nist.gov/srmext/certificates/1648a.pdf), the extended categories were analysed to expand the applicability of the ExposomeMap-PM model.

Categories of particles are reviewed in Table 1 based on previous works that included descriptions of particle types, their composition and toxicological mechanisms.

**TABLE 1:** Types of particles, their sources, features and related toxicological mechanisms and outcomes.

Particle type/composition	Sources	Size range	Mechanism	Receptor/Initiator	Key event
Elemental carbon/black carbon (soot)	Diesel exhaust, gasoline exhaust, coal fly ash, wildfire/biomass smoke	UFP, fine	Scavenger-receptor uptake of carbon core	MARCO, MSR1, CD36, lysosomal stress, TLR4	Phagocytic uptake, NLRP3 priming, ROS
Organic carbon/PAHs/quinones (oxy-PAH)	Diesel/gasoline exhaust, cooking, wildfire smoke	UFP, fine	PAH ligand binding; quinone redox cycling	AHR, CYP1A1/CYP1B1, NOX2	AHR activation, ROS generation
Metals (Fe, Cu, Ni, Zn, Pb, Cd, Cr, Mn)	Brake wear, welding UFP, zinc UFP, industrial manufacturing, road dust, coal fly ash	UFP, fine, coarse	Surface Fenton/redox cycling	Fenton chemistry (Fe2+/Fe3+, Cu+/Cu2+), Cr6+, Cr3+; TRPM2; NOX2	ROS, oxidative stress
Crystalline/mineral silica (SiO_2_), aluminosilicates	Silica, road dust, construction, mining, desert and dust-storm dust, volcanic ash, agricultural tilling and dust, granite/marble sanding	UFP, fine, coarse	Scavenger uptake, lysosomal membrane damage, inflammasome	MARCO, MSR1 (SR-A1/CD204), CD36, endocytosis, lysosomal damage, NLRP3, IL1B/IL18	Lysosomal destabilisation, NLRP3 inflammasome, oxidative burst
Secondary inorganic ions: sulfate, nitrate, ammonium	Secondary from SO_2_ (coal/industry), NOx (vehicles/power), NH_3_ (agriculture)	fine	Acidity, hygroscopic growth, solubilisation of co-particle metals	No direct receptor; alters metal solubility, Fenton	Modifier of metal bioavailability: Raises solubility/ROS of co-particles
Secondary organic aerosols (SOA)	Atmospheric photochemistry	UFP, fine	Reactive oxygenated organics/electrophiles	ROS; partial AHR	Oxidative stress
Bioaerosols/endotoxin (LPS), microbial fragments	LPS particles, agriculture, wastewater, composting, carried by tilling/drought and desert dust	fine, coarse	PAMP recognition	TLR4 (+CD14/MD2), TLR2, MYD88, NF-κB	TLR activation, NF-κB inflammation
Engineered nanoparticles (TiO2, SiO2, Ag, CNT)	Carbon nanotubes, industrial nanomaterial production	UFP	High surface-area reactivity; frustrated phagocytosis (fibres)	Scavenger receptors, NLRP3 (CNT)	ROS, NLRP3, frustrated phagocytosis

### Related diseases

This task used PubMed, following the search approach described at the start of the Methods. Example search queries:

“Particulate Matter” AND disease AND pathway,

“Particulate Matter” AND disease AND mechanisms,

“Particulate Matter”[Mesh] AND disease[tw] AND pathway[tw].

The term “disease” was replaced by specific terms during the search process, for example:

“Particulate Matter” AND Alzheimer’s disease AND mechanisms.

### Determining architecture and scope

The architecture and scope of the ExposomeMap-PM were defined following the logic of the integrated AOP network (Figure 2), with a deliberate focus on the earlier molecular and cellular events that precede organ-level outcomes. Building on the integrated AOP framework, relevant pathway-level mechanisms were reviewed to estimate the size of the model and the key blocks to include to cover particle-induced effects across respiratory, cardiovascular and neurological systems. These were then merged and harmonised at the stage of developing a connectivity table (see the following sections). This step established the map’s overall structure, system coverage, and level of granularity, including the selection of representative molecules, receptors, and cell types.

### Reviewing publications on relevant mechanisms

To identify relevant mechanisms and specifically receptors involved, we searched PubMed for recent reviews using such terms as “fine particle”, “ultrafine particles” or “particulate matter” in combination with “receptor” in titles and abstracts. We then extracted the original research articles cited in the reviews to obtain detailed information about relevant receptors. The list of references used for building the ExposomeMap-PM is available in Supplementary Material S2.

### Developing connectivity table

The main connectivity table (Supplementary Material S3) is designed as an intermediate step for 1) identifying key cell types and molecules involved, 2) identifying entities by linking them to external databases, 3) making it easy for domain experts to contribute to the resource development by adding information. The connectivity table and map were curated by the development team, with individual contributors responsible for specific systems and mechanism areas and domain experts reviewing the corresponding content. The resource is structured for open review: the connectivity table allows domain experts to inspect and correct entries, and the published map has MINERVA commenting functionality for continued community review and updates.

### Developing diagram in CellDesigner

The ExposomeMap-PM model was constructed in CellDesigner using the notation compliant with Systems Biology Graphical Notation (SBGN) standard (Le Novère et al., 2009) to represent molecular interactions underlying particle-induced toxicity. Reactions, complexes, and compartments were defined according to the disease-map curation guide (Mazein et al., 2023). Molecular reactions were built from primary literature and cross-checked against established pathway resources, in particular the Reactome and KEGG pathways for reactive oxygen species detoxification (Reactome:R-HSA-3299685) and reactive oxygen species (KEGG:hsa05208), as marked on the map.

### Publishing *via* the MINERVA platform

The curated CellDesigner model was then made available online *via* the MINERVA Platform with the corresponding automatic annotation for map entities to facilitate exploration, reuse and community curation. The map entities were automatically annotated during MINERVA upload using MIRIAM-compliant identifiers. The MINERVA Platform provides zooming and navigation, enabling uploading omics data to create overlays for visualisation and interpretation. Export functions allow snapshot generation and map extraction in various formats including CellDesigner’s XML, SBGN-ML, image formats, table of entities and table of interactions. The map was released under the Creative Commons Attribution 4.0 International License (CC BY 4.0) with built-in commenting capabilities for collaborative updates.

### FAIR principles

The ExposomeMap-PM was developed and made available as a FAIR (Findable, Accessible, Interoperable and Reusable) resource, following the criteria established (Balaur et al., 2025; Mazein et al., 2023). The underlying MINERVA Platform addresses several elements of FAIR (Balaur et al., 2025). Findability is supported by a version-specific Zenodo DOI as a persistent identifier for the archival snapshot, with each release deposited as a new version under a shared concept DOI, and by annotating by connecting entities to well-established biomedical resources such as HGNC, UniProt, ChEBI and PubMed using standard identifiers. Accessibility is ensured by online access to the map content and authentication procedures where necessary. Interoperability is supported by the use of systems biology standards such as SBML and SBGN formats, which can be translated to other standard formats *via* the MINERVA converter. Reusability is facilitated by release under the Creative Commons Attribution 4.0 International License (CC BY 4.0), by annotation with stable identifiers for molecular entities (UniProt, ChEBI) and by commenting capabilities that support community curation. Following these principles ensures the long-term reuse of the resource and its value to the systems toxicology and biomedicine communities.

### Data visualisation

Differential expression values from transcriptomics and proteomics datasets were visualized on the ExposomeMap-PM using the MINERVA Platform. Because the datasets are mouse-derived and the map entities carry human identifiers, overlay values were matched to node HGNC symbols by gene symbol, case-insensitive. Mouse symbols with no matching node were not overlaid. Node symbols were then checked against mouse orthologs using HGNC HCOP (https://www.genenames.org/tools/hcop/) (Yates et al., 2021). 143 of the 144 HGNC-annotated nodes (99.3%) have a mouse ortholog and 139 (96.5%) share the same symbol. The exceptions are CHI3L1 (Chil1), TP53 (Trp53), TXN (Txn1), CXCL8 (weak, Cxcl15) and NOX5 (no mouse ortholog). The full mapping is in Supplementary Material S4. Differential expression was computed using limma-voom for transcriptomics (Law et al., 2014) and ProDA for proteomics (Ahlmann-Eltze and Anders, 2020). Only features significant at nominal p < 0.05 in the corresponding analysis were retained for overlay. Features above this threshold were displayed. Nominal p was used in place of a Benjamini–Hochberg-adjusted threshold. The testing family that BH correction is calibrated to differs from the family in which the claim is made. BH q-values in these datasets are computed across the full transcriptome and proteome, whereas the overlay interrogates a small, literature-curated, *a priori* node set selected before any data were examined. Fold-change values were uploaded as overlay files, automatically mapped to corresponding protein and colour-coded by direction and magnitude of change. Log2 fold-changes were linearly rescaled in the uploaded overlay files to the interval ±0.8 for colour mapping in the MINERVA Platform where colour scale is fixed from −1 (full red) to +1 (full blue). Rescaling to ±0.8 instead of to the full ±1 keeps values just below full saturation. This way, the node fill does not obscure the node labels. The rescaling is linear and preserves the order and relative magnitude of the values. It affects display only, not the values or their interpretation. The legend for colour intensity is available in the overlays tab in MINERVA. Dataset-specific details including exposure conditions and acquisition parameters are described in the “Results” section.

To quantify the extent and concentration of the overlays, the complete T1 and P1 feature tables were retained as the testing background, unique gene symbols were counted once, and nominal differential expression was defined as p < 0.05, matching the overlay display rule. Map membership was assessed against the 144 HGNC-annotated nodes listed in Supplementary Material S4. For each dataset, 10,000 random gene sets of the same size as the represented map-node set were drawn from the complete tested background and the number of nominally differentially expressed genes was compared with the observed value to obtain an empirical permutation p-value and a fold-enrichment. Block-level differential-expression fractions, mean absolute log fold-changes and one-sided hypergeometric p-values against the same background are reported in Supplementary Material S5.

A step-by-step guide for data uploading and creating customised overlays is available at https://disease-maps.io/markopolo/overlays. Researchers can create their own account and securely experiment with their data using the MINERVA Platform.

## Results

### Integrated adverse outcome pathway network

Following the established methodology (Mazein et al., 2025, 2024), the adverse outcome pathway (AOP) concept was used to integrate available AOPs related to particle-induced toxicity, and then, building on this foundation, to develop a detailed mechanistic representation within the disease map concept. This merged approach allows a systematic and consistent translation from physiological-level context of AOPs to a molecular-level representation of a disease map, this way providing a framework for representing toxicity mechanisms.

Based on the previously published AOPs, an integrated AOP network was developed (Figure 2) (Halappanavar et al., 2020; Li T et al., 2022; Yu et al., 2024; Zhang et al., 2024). This network represents epidemiological association to possible outcomes - pathophysiological phenotypes and diseases. The AOP network consolidates pathways linking particle exposure to respiratory, cardiovascular and neurological toxicity by aligning overlapping key events across initial AOPs. Redundancy is reduced by merging conceptually similar elements such as oxidative stress, inflammation, mitochondrial dysfunction, apoptosis and altered gene expression. The integrated AOP network provides a systems-level view of toxicity across the three systems.

### Particle types: source, chemical composition, size

Airborne particles originate from natural sources (dust storms, volcanic eruptions, sea spray, forest fires, pollen), combustion sources (vehicle exhaust, power plants, residential heating, industrial boilers), industrial processes (cement production, metal smelting, chemical manufacturing, mining), agricultural activities (plowing, harvesting, fertilizer application, livestock emissions), as well as secondary formation in the atmosphere (particles formed from gaseous precursors like sulfur dioxide and nitrogen oxides), household sources (cooking, smoking, candles, cleaning sprays, air conditioning), road dust and wear particles (tire and brake wear, road surface abrasion), marine and water sources (sea spray aerosols, microplastics), and biological sources (bacteria, viruses, spores, plant or animal fragments) (Kim et al., 2015; Srimuruganandam and Shiva Nagendra, 2012).

Particles can also be classified by chemical composition, including water-soluble secondary aerosols such as sulfates (from SO2), nitrates (from NOx), ammonium salts (NH4+ combined with sulfate/nitrate, common in agricultural regions), organic carbon (volatile and semi-volatile organics from combustion and biomass burning), elemental carbon (black carbon/soot from incomplete combustion), metals and trace elements (lead, cadmium, iron, copper, zinc, nickel from industry and road dust), mineral dust (silica, alumina, calcium compounds from soil or construction sites), sea salt and chloride particles (NaCl from sea spray), biogenic particles (pollen, plant waxes, microbial fragments), and secondary organic aerosols (formed in the atmosphere from reactions of volatile organic compounds) (van Donkelaar et al., 2019).

Particle size is linked to particle source and composition. UFPs mostly consist of combustion and secondary formation particles such as organic carbon, elemental carbon, and secondary organic aerosols (Sioutas et al., 2005). PM2.5 includes these components, as well as fine particulate sulfates, nitrates, ammonium salts, and some fine natural dust from sources such as forest fires, volcanic ash, or dust storms (Thangavel et al., 2022). Larger particles (>2.5 µm) are primarily from mechanical and natural processes, including road dust, construction dust, sea spray, and pollen (Ribeiro et al., 2015; Steerenberg et al., 2006).

For the map presented here, we need to distinguish different types of particles based on their chemical composition and particle size. Chemical composition determines the mechanisms by which they can trigger biological pathways. This includes sulfates, nitrates, ammonium salts, organic carbon, elemental carbon, metals and trace elements, mineral dust, sea salt, biogenic organics, and secondary organic aerosols. Particle size influences how particles enter the body and which tissues or organs they can reach. Ultrafine particles (UFPs, ≤0.1 µm) can penetrate deep into the lungs and even enter the bloodstream (Geiser et al., 2005). The smallest fraction of UFPs can deposit in the nasal olfactory region and subsequently translocate along the olfactory nerve towards the brain (Lintusaari et al., 2025). Fine particles (PM2.5, 0.1–2.5 µm) deposit in the lower respiratory tract and include both combustion and secondary inorganic or organic components (Schlesinger, 2007). Larger particles (>2.5 µm) mostly deposit in the upper airways or remain near the site of exposure, typically originating from mechanical, biological, or natural sources (Deng et al., 2019).

In terms of ways particles induce biological effects, there are three main mechanisms:

1. Direct receptor interactions–particles or particle-bound chemicals can act as ligands for cell-surface or intracellular receptors, e.g., Toll-like receptors, aryl hydrocarbon receptors, triggering signalling pathways and inflammatory responses.
2. Oxidative stress–many particles, especially those containing metals, elemental carbon, or reactive organics, can generate reactive oxygen species (ROS) either on their surface or inside cells, leading to oxidative damage, activation of transcription factors (like NF-κB), and secondary inflammatory cascades.
3. Ion channel modulation–some particles, particularly ultrafine or metal-containing ones, can affect calcium (Ca2+) signalling in cells, either by disrupting membranes or interacting with ion channels, which influences processes like apoptosis, secretion, and contraction.

### Diseases associated with particle-induced toxicity

Exposure to PM2.5 and UFPs is associated with a wide spectrum of chronic diseases across multiple systems. Epidemiological and mechanistic studies link particle exposure to respiratory, cardiovascular, neurological, dermatological, autoimmune, musculoskeletal, and gastrointestinal pathologies. Shared molecular mechanisms - particularly oxidative stress, inflammation, endothelial dysfunction, mitochondrial impairment, and immune dysregulation - contribute to these outcomes. The diseases summarized in Table 2 reflect both direct tissue-level toxicity and indirect systemic responses, as shown by integrated toxicological and systems biomedicine studies. Except for well-characterised crystalline silica and silicosis exposure with confirmed causation (Marrocco et al., 2021), the relationships depicted in Table 2 are presented as associations, and confirmed related mechanisms and outcomes that depend on exposure level, duration and related pre-existing health conditions.

**TABLE 2:** Examples of diseases relevant to PM2.5 and UFP exposure.

Disease/Outcome	Disease ID	Key mechanisms	References	Evidence type
Cardiovascular				
Ischemic heart disease	DOID:5844	Coronary oxidative stress and endothelial dysfunction, cardiomyocyte injury, and pulmonary sensory receptor–driven autonomic/adrenergic imbalance contribute to ischemic cardiac dysfunction.	(Brook, 2008; Miller, 2020; Robertson et al., 2014)	r-exp; review
Cerebrovascular diseases	DOID:6713	Oxidative stress and vascular inflammation drive cerebrovascular dysfunction and ischemic injury *via* NLRP3 inflammasome activation; endothelial dysfunction and IL-6-mediated fibrinogen upregulation sustain a prothrombotic state leading to cerebral ischemia.	(Chen et al., 2022; Li W et al., 2022; Rubio et al., 2025; Scheers et al., 2015)	Meta; review
Hypertension	DOID:10763	Vascular oxidative stress and autonomic imbalance elevate arterial pressure and stiffness; ROS-mediated RhoA/ROCK activation potentiates vasoconstriction; PM-induced DNA hypomethylation activates inflammatory and vascular pathways.	(Li et al., 2017; Mills et al., 2009; Ying et al., 2009)	h-exp; m-exp; review
Heart failure	DOID:6000	Endothelial dysfunction, autonomic imbalance, and pro-thrombotic shifts activate redox-sensitive pathways (NF-κB, Nrf2) and inflammasome signalling (NLRP3), driving mitochondrial dysfunction, impaired myocardial contractility, and arrhythmogenic remodelling.	(Brook, 2008; Münzel et al., 2020; Weldy et al., 2014)	m-exp; review
Heart conduction disease	DOID:10273	Autonomic dysregulation and oxidative stress disrupt cardiac conduction *via* carotid body ion channel dysregulation (Na+/K+ channels), SCN5A nitration, and TRPA1-mediated sympathetic activation.	(Bevan et al., 2021; Fiordelisi et al., 2017; Hazari et al., 2011; Mandaglio-Collados et al., 2023)	m-exp; r-exp; review
Atherosclerosis	DOID:1936	Endothelial activation, macrophage lipid uptake and foam cell formation drive plaque instability; ROS-mediated aortic oxidative stress and impaired HDL protective capacity accelerate atherosclerotic progression.	(Araujo et al., 2008; Bevan et al., 2021; Seneviratne and Miller, 2025; Sun et al., 2005)	m-exp; review
Chronic kidney disease	DOID:784	Endothelial dysfunction, ROS-induced podocyte injury, and microvascular inflammation; GPX4-regulated ferroptosis and epithelial-to-mesenchymal transition in tubular cells.	(Bowe et al., 2018; Hsu et al., 2019; Lin et al., 2025)	h-epi; r-exp; h-in vitro
Lung disease				
Pneumonia	DOID:552	PM exposure downregulates antimicrobial peptides (α-defensin, S100A7, hBD-2) *via* ROS-mediated epithelial senescence, impairing airway antibacterial defence and promoting bacterial invasion.	(Chen et al., 2018; Piyadasa et al., 2018)	h-exp; h-in vitro
Pulmonary fibrosis	DOID:3770	Macrophage lactate production stabilises TGF-β1 *via* CHIP suppression, driving epithelial-to-mesenchymal transition and lung fibrosis.	(Liu et al., 2025; Wang et al., 2025)	m-exp; m-in vitro; h-in vitro
Asthma	DOID:2841	Epithelial injury and Th2-mediated inflammation amplify bronchial hyperreactivity and remodelling; EGFR-PI3Kα-AKT/ERK signalling drives mucus hypersecretion; DEP-induced CXC chemokines recruit neutrophils and augment airway inflammation.	(Fortoul et al., 2011; Kim et al., 2011; Xu et al., 2020)	m-exp; review
COPD	DOID:3083	Chronic oxidative stress and inflammation cause airway remodelling and emphysema; NF-κB/Nrf2 pathways and downstream cytokine, mucus, and remodeling responses.	(Cachon et al., 2014; Fortoul et al., 2011; Kaur et al., 2022)	h-in vitro; review
Lung cancer	DOID:1324	DNA damage, chronic inflammation, and epigenetic dysregulation; DEP-induced immunosuppressive tumor microenvironment promoting KRAS-driven adenocarcinoma progression.	(Delhez et al., 2026; Fortoul et al., 2011; Hill et al., 2023; Valavanidis et al., 2013)	h-epi; m-exp; review
Neurological disorders				
Alzheimer’s disease	DOID:10652	Chronic exposure induces oxidative stress, microglial activation, gliosis, amyloid pathology, tau aggregation, mitochondrial dysfunction, and excitotoxicity.	(Liu et al., 2024; Olloquequi et al., 2024; Peters, 2023; Rubio et al., 2025; Sahu et al., 2021)	m-exp; review
Parkinson’s disease	DOID:14330	Neuroinflammation, oxidative stress, and α-synuclein fibrillization drive dopaminergic neuronal loss; CpG hypermethylation silences PINK1/DJ-1; suppressed mitophagy promotes mitochondria-mediated apoptosis.	(Calderón-Garcidueñas et al., 2020; Du et al., 2025; Rubio et al., 2025; Wang et al., 2021; Yuan et al., 2022)	m-exp; r-exp; *in vitro*; review
Cognitive impairment & dementia	DOID:1561	Chronic neuroinflammation, oxidative stress, microvascular compromise, and hippocampal neurogenesis inhibition; BACE1 upregulation promotes amyloidogenic APP processing.	(Rubio et al., 2025; Wilker et al., 2023)	Meta; review
Lewy body dementia	DOID: 12217	Alpha-synuclein aggregation and propagation, neurodegeneration, brain atrophy, and cognitive decline.	(Zhang et al., 2025)	h-epi; m-exp; *in vitro*
Attention deficit hyperactivity disorder	DOID:1094	Microglial hyperactivation, dopamine-pathway disruption, impaired myelination and oxidative stress affecting fronto-striatal circuit development.	(Ahmad et al., 2024)	Meta
Autism spectrum disorder	DOID:0060041	Neuroinflammation, glial activation, synaptic pruning disruption, mitochondrial dysfunction, and epigenetic shifts impair cortical circuits; oxytocin reduction across limbic, prefrontal, and cerebellar regions contributes to social behaviour deficits.	(Dutheil et al., 2021; Rahmatinia et al., 2024)	r-exp; meta

Disease Ontology database (https://disease-ontology.org) was used for identifying diseases, for example for Parkinson’s disease it is DOID:14330 (https://disease-ontology.org/?id=DOID:14330) and from there can be linked to other disease databases such as ICD10CM, ICD9CM, MESH and OMIM. An extended version of the table is available in Supplementary Material S6 and includes infectious, skin, metabolic, autoimmune, gastrointestinal and reproductive diseases. Evidence type: h, human; m, mouse; r, rat; epi, epidemiological; exp, *in vivo* experimental; *in vitro*, *in vitro* experimental; review, review article; meta, systematic review/meta-analysis.

### ExposomeMap-PM resource design

In the initial stage of model development, we focused on the first-wave cellular responses - those occurring in direct contact with inhaled particles, while limiting the number of cell types represented to ensure mechanistic clarity and model feasibility. This level captures the earliest molecular initiating events and primary key events in alveolar and airway epithelium, macrophages and at endothelial interfaces, which collectively define the onset of oxidative, inflammatory, and barrier-related perturbations. Focusing on these core interactions allows mapping of causally interconnected molecular pathways before introducing additional complexity. Accordingly, the downstream signalling is represented within a generalised cellular context that does not yet capture cell-type-specific context. The framework is designed for modular expansion, enabling future integration of second-wave processes and additional cell types across respiratory, cardiovascular, and neurological systems. The framework is designed in a way so that extensions are built on the existing nodes, with new cell type context and second-wave processes added as further layers of information that refine and expand the map as the resource grows. The resource is to be maintained through a continued curation cycle, with the map reviewed and updated, including existing content, as new evidence becomes available.

ExposomeMap-PM version 1.0 includes 304 entities and 497 processes. The entities include 144 proteins, 25 genes and 24 mRNAs, 43 simple chemicals, 10 complexes, 35 phenotypes and 23 particle sources and types. 214 of 236 molecular nodes (91%) have MIRIAM-compliant identifiers linked to HGNC, UniProt, Ensembl, NCBI Gene and ChEBI, and the entities without annotation are generic family or complex placeholders and reactive or transient chemical species. A complete quantitative breakdown is provided in Supplementary Material S7.

To follow the AOP-type logic illustrated in Figure 2 - from initial interactions of particles with cells to the system-level outcomes and conditions affected - and to account for different levels of complexity, the following considerations were included. To enable systematic representation of the mechanistic landscape of fine and ultrafine particle toxicity, we established a structured framework that connects physicochemical particle properties to molecular initiating events (MIEs), cellular responses, and disease outcomes. This framework integrates multiple dimensions including:

‒ particle composition,
‒ particle size,
‒ interaction type,
‒ target cell specificity,
‒ receptor or ion channel engagement,
‒ immediate gene-level consequences,
‒ propagation of secondary-wave effects,
‒ resulting physiological and pathological endpoints.

By organizing these elements, the approach makes it possible to reflect the complexity while keeping the model manageable.

Another important aspect is that, while ExposomeMap-PM introduces varying levels of conceptual complexity, it consistently follows the Systems Biology Graphical Notation (SBGN) standard, using its elements to represent knowledge. For example, early molecular events are presented using the main elements (macromolecules and simple chemicals). At the same time, more abstract entities - such as physiological and pathological endpoints - are represented using phenotype elements. This enables compatibility with other systems biology resources and tools, as well as computational modelling.

### Molecular initiating events and their outcomes

At the molecular level, particle-induced toxicity begins with a set of molecular initiating events (MIEs) triggered by the physical and chemical properties of fine and ultrafine particles. These MIEs involve direct interactions with cellular receptors, ion channels, and membranes that activate redox-sensitive and inflammatory signalling cascades. These events lead to downstream key events such as oxidative stress, inflammasome activation, cytokine release, apoptosis, and autophagy, that collectively drive the mechanisms represented in the ExposomeMap-PM. Table 3 shows an excerpt from a more extensive connectivity table (Supplementary Material S3) focusing on receptors to provide a structured overview of the initiating molecular interactions underlying particle-induced systemic toxicity. This connectivity table (Supplementary Material S3) serves as a format for refining the content of the ExposomeMap-PM and for communicating with experts to systematically list known initiating events.

**TABLE 3:** A fragment of the connectivity table (Supplementary Material S3) describing the involvement of the SR-A1 (MSR1) receptor in mechanisms induced by fine and ultrafine particles.

Field	Content
Receptor/mechanism	SR-A1/CD204
HGNC name	MSR1
Particle type	Silica-rich and metal-rich PM2.5/UFP
Particle source	Dust, construction, industry
Particle chemistry	Crystalline silica, iron oxides
Particle size range	Coarse, PM2.5, UFP
Interaction type	Scavenger-mediated uptake
First-wave target cells	Alveolar macrophages
Pathway	Silica/metal PM > MSR1 > endocytosis > lysosomal damage > NLRP3 inflammasome; MSR1 > endocytosis > CpG-DNA > MYD88 > IRF7 > IFNA/B
Genes activated (first-wave)	NLRP3, IFNA, IFNB, IL1B, IL18
Secondary-wave propagation	Neutrophil recruitment, cytokine release, antiviral effects
Connected diseases/pathological endpoints	Pulmonary fibrosis
References	PMID:36325338, PMID:24941076, PMID:17361018

The connectivity table serves as an initial framework for developing a map of relevant mechanisms. Supplementary Material S3 contains 17 receptor or initiating-mechanism records.

### Graphical model

The interconnected direct and indirect mechanisms (Table 3, Supplementary Material S3) are translated into a graphical representation of the ExposomeMap-PM (https://disease-maps.io/markopolo), readable by both humans and machines. Figure 3 shows a fragment of the ExposomeMap-PM including receptor-mediated particle recognition and uptake, downstream signalling, and ROS-related mechanisms. The fragment illustrates connections from particle types through scavenger receptors and endocytosis to intracellular signalling pathways and activated genes.

**FIGURE 3:**
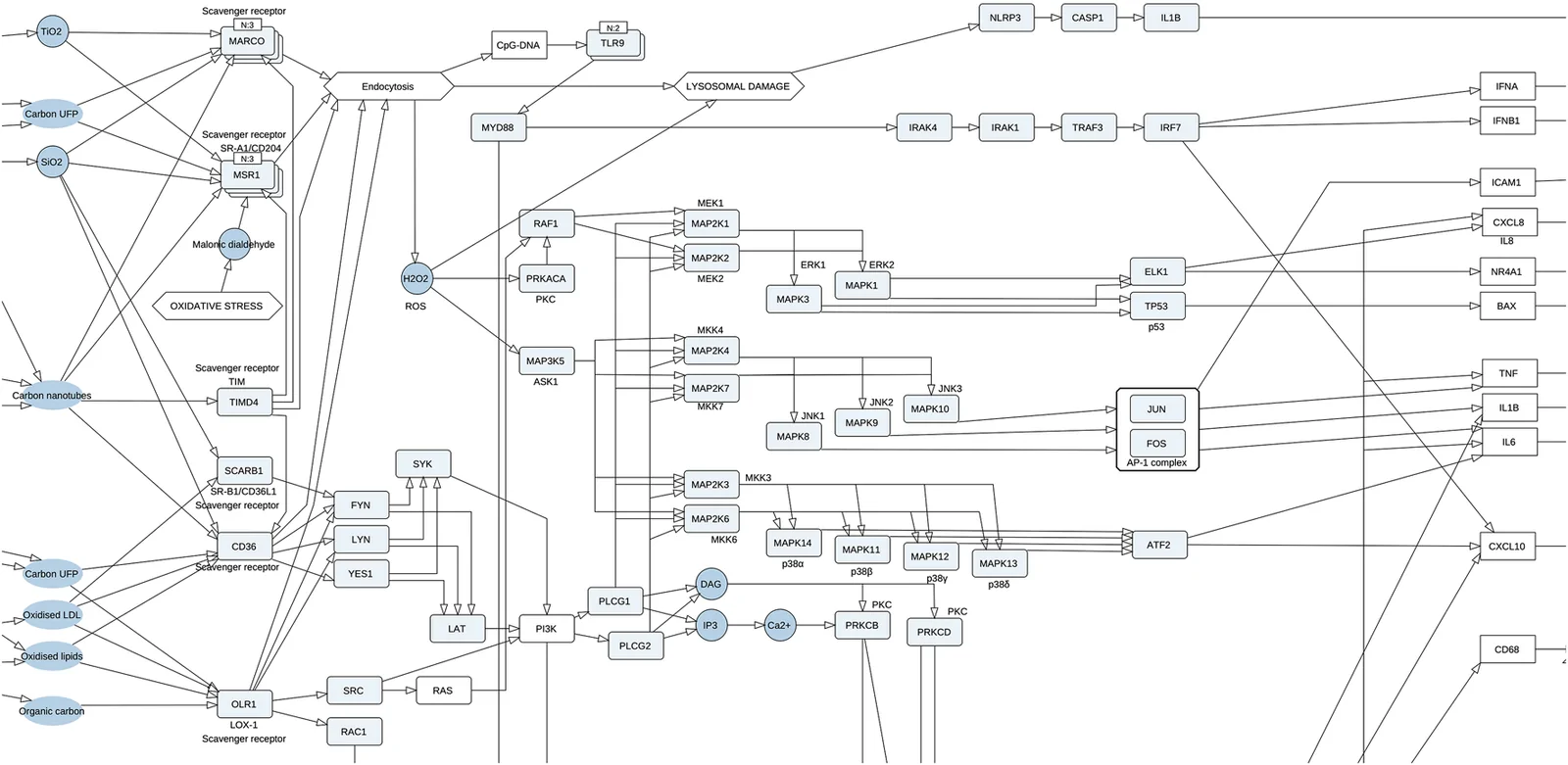
A fragment of the ExposomeMap-PM model in the MINERVA Platform. The figure shows a fragment of the map as it appears in the interactive platform, where connections are explored by browsing and querying, and it is intended to convey the overall structure and flow of information. The flow of information from left to right: particle sources > particle types > receptors or receptor-independent mechanisms > signalling pathways > genes activated > phenotype outcomes > connected diseases. The connectors to phenotype outcomes and to diseases show proposed mechanistic links and functional association and do not establish direct causality. The five mechanistic blocks represented in the map are described in the “Blocks of the ExposomeMap-PM” section. The complete ExposomeMap-PM is accessible online for browsing and exploration *via* https://disease-maps.io/markopolo. PDF version is provided in Supplementary Material S8.

The ExposomeMap-PM model details how fine- and ultrafine-particle exposure triggers a continuum of molecular perturbations that propagate from receptor or ion-channel engagement to organ-level pathology.

### Blocks of the ExposomeMap-PM

The map is organised into five mechanistic blocks. A colour-coded overview version of the map is accessible *via* https://disease-maps.io/markopolo and is also provided in PDF format in Supplementary Material S9. The five blocks represent groups of the early molecular events triggered by particle deposition on epithelial and immune cells and the responses these events provoke. These five blocks refine the three main mechanisms introduced earlier: direct receptor interactions correspond to receptor activation and to phagocytic recognition, oxidative stress corresponds to the surface-driven catalytic chemistry and the antioxidant response that counters it, and ion channel modulation corresponds to the ion channel perturbation block.

#### Receptor activation as primary sensors of particle exposure

TLR2 and TLR4 are the first-line innate immune sensors of particles. TLR2 can make heterodimers with TLR1 or TLR6, while TLR4 forms a complex with MD2 and CD14. Downstream they converge on TIRAP-MYD88 and activate pro-inflammatory IRAK4/IRAK1 and TAK1-IKK-NF-κB transcription and TRAF3-IRF7 type I interferon events. TAK1 also activates JNK and p38 MAPK cascades through to AP-1.

#### Receptor-mediated uptake and phagocytic recognition

Scavenger receptors MARCO and MSR1 as well as CD36, OLR1 and SCARB1 bind carbon and mineral particles and drive their internalisation. Particle accumulation in lysosomes causes lysosomal damage that activates NLRP3 and IL1B. CD36 and OLR1 activate Src-family kinases FYN and LYN which signal through PI3K-PLCG to PKC isoforms.

#### Ion channel perturbation and membrane signalling disruption

TRP channels TRPV1, TRPV4 and TRPA1 are the main sensors of particle-induced mechanical and oxidative stress. TRPM2, TRPM8 and TRPC4 contribute to Ca2+ influx. Ca2+ entry activates calmodulin-CaMKK-CaMK2 signalling into RAF-MEK-ERK, drives PKC-dependent NF-κB activation, promotes ROS generation and epithelial barrier disruption. ROS sensitises TRPV3 and TRPV4, creating a positive feedback loop between oxidative stress and calcium dysregulation.

#### Oxidative and catalytic interactions at the particle surface

Transition metals released from particles generate hydroxyl radicals through Fenton chemistry, with Fe2+/Fe3+ and Cu+/Cu2+ redox cycling as the key reactions. Chromium undergoes sequential reduction from Cr6+ to Cr3+ and produces hydroxyl radicals at each step. Polycyclic aromatic hydrocarbons activate AHR with downstream activation of CYP1A1 and CYP1B1 and generation of ROS. NOX2 is the main enzymatic superoxide source. It is activated downstream of scavenger receptors and PKC signalling.

#### Antioxidant and detoxification response

This part represents the enzymatic defense against particle-induced ROS. It includes superoxide dismutases SOD1/2/3, catalase, glutathione peroxidases, peroxiredoxins, thioredoxins and NQO1. NRF2 drives transcription of this defence pathway. The balance between detoxification ability vs. ROS production determines whether oxidative stress results in downstream effects.

These five blocks result in a shared set of downstream pathophysiological effects and are connected to related diseases. Particle specificity resides at the molecular-initiating-event level, while the downstream cascades are shared convergence points. Examples include ROS-driven modifications of redox-sensitive regulators such as NRF2 and NF-κB, inflammasome assembly, and mitochondrial dysfunction linked to the inflammatory and apoptotic changes downstream on the map.

### Omics data visualisation

Figure 4 shows an example of omics data visualisation using the MINERVA Platform (https://disease-maps.io/markopolo). Differential-expression values from the transcriptomics and proteomics datasets are overlaid onto the protein/mRNA/gene nodes of the ExposomeMap-PM, providing a pathway-level view of the particle-induced effects. Upregulated entities are displayed in blue and downregulated entities in red, with the color intensity proportional to the magnitude of the fold change. This visual encoding enables visualisation of differential-expression values, making condition-specific molecular responses immediately interpretable within their mechanistic context. The transcriptomics dataset (T1) is from Kuntic et al. (2023), from the PM-exposure arm (n = 47) compared against the clean-air control arm (n = 43) of the same experiment. The noise-only and PM-plus-noise arms were not used, so the contrast contains no noise co-exposure component. Mice were exposed for 6 h per day for 3 days to ambient PM with a concentration of 200 μg/m^3^ and mRNA in lung tissue was quantified. This exposure is an acute high-concentration challenge, selected to elicit a molecular response of sufficient magnitude to illustrate the overlay functionality. It exceeds the WHO 2021 global air quality guideline values for PM2.5 by more than an order of magnitude and is well above typical urban ambient concentrations, although it is within the range of short-term peaks recorded during severe pollution episodes in heavily polluted urban areas. It should therefore be read as a proof-of-principle dataset for data integration, representing a controlled acute exposure that sits at the high end of the real-world range. The proteomics dataset (P1) was generated by performing bottom-up untargeted data independent acquisition (DIA)-based proteomics on lung tissue from the same two arms (n = 5 per group). Briefly, proteomic raw files were processed using Spectronaut v20.0 to generate protein-level quantification results and were used for disease-map integration alongside T1. For a detailed description of the proteomics acquisition, processing, and results, please refer to Rogowska-Wrzesinska and coauthors (dataset, https://doi.org/10.5281/zenodo.18832608). For both datasets, gene names were mapped to gene symbol notation by using UniProt ID Mapping. Differential expressions of transcripts and proteins were performed by using limma-voom (Law et al., 2014) and ProDA (Ahlmann-Eltze and Anders, 2020) respectively.

**FIGURE 4:**
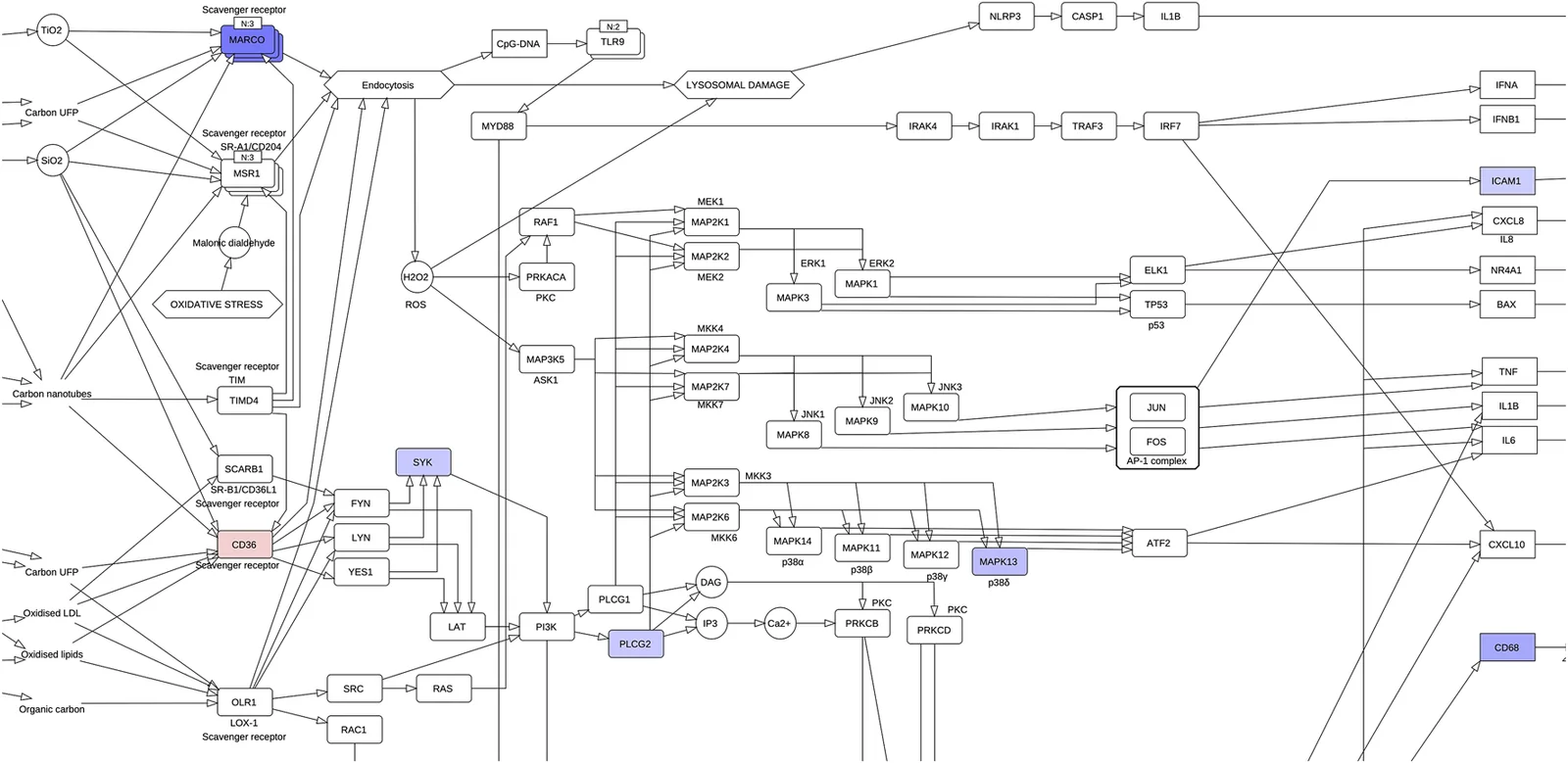
A fragment of the ExposomeMap-PM map with P1 omics dataset visualisation. The fragment demonstrates how experimental measurements are projected directly onto the map’s nodes, so that condition-specific molecular responses can be read within their mechanistic context. Only features significant at nominal p < 0.05 are displayed; upregulated entities are shown in blue and downregulated in red, with colour intensity proportional to the fold change. Log2 fold changes were linearly rescaled to ±0.8 for display in the MINERVA colour scale. Interactive browsing and data visualisation is accessible *via* https://disease-maps.io/markopolo. To see the datasets, click the “Overlays” button on the top of the MINERVA Platform interface.

To assess how well the model captures the measured response, we quantified the concentration of nominally differentially expressed features (p < 0.05) among the map nodes represented in each complete feature table (Table 4). Of the 144 HGNC-annotated nodes, 131 were represented among the 18,606 unique symbols tested in T1 and 79 among the 5,739 tested in P1, so most of the model is directly addressable by these datasets. Against 10,000 random gene sets matched to the represented map-node count and drawn from the same tested background, the T1 map-node set was significantly enriched for differentially expressed genes (35 observed *versus* a mean of 23.30, 1.50-fold, permutation p = 0.0074), and the P1 map-node set pointed in the same direction (18 observed *versus* 15.97, 1.13-fold, p = 0.3199). All five mechanistic blocks contained differentially expressed nodes in both datasets, with fractions of 19.6%–38.5% in T1 and 17.6%–33.3% in P1; the highest fractions in T1 were in the ion channel perturbation (38.5%) and antioxidant and detoxification (30.8%) blocks, consistent with the mechanisms expected to be engaged first (Supplementary Material S5). Individual block tests are inherently underpowered at these block sizes, which range from 3 to 56 represented nodes, and are reported for completeness rather than as independent evidence. This is the expected picture for a proof of concept based on a single reference particle type and a short, 3-day exposure: the transcriptomic response is already measurably concentrated on the map, and the proteomic response, quantified over a shallower background and subject to the delay between transcript and protein change, follows the same trend. Longer exposures, additional particle types and deeper proteome coverage are the natural next step for resolving effects block by block, and ExposomeMap-PM provides the mechanistic frame in which such datasets can be projected and compared.

**TABLE 4:** Mapping and enrichment of the overlays on ExposomeMap-PM nodes.

Metric	T1 transcriptomics	P1 proteomics
Unique gene symbols tested (complete table)	18,606	5,739
ExposomeMap-PM nodes represented	131	79
Differentially expressed features (p < 0.05)	3,312	1,160
Differentially expressed map nodes	35	18
Differentially expressed fraction of represented nodes	35/131 (26.7%)	18/79 (22.8%)
Matched random-set mean (SD)	23.30 (4.38)	15.97 (3.54)
Enrichment	1.50-fold	1.13-fold
Empirical permutation p-value	0.0074	0.3199

Differential expression is defined as nominal p < 0.05 in the complete feature table of each dataset; unique gene symbols were counted once. Enrichment is the ratio of the observed number of differentially expressed map nodes to the mean of 10,000 size-matched random gene sets drawn from the same tested background, and the p-value is the corresponding empirical permutation p-value.

## Discussion/outlook

The ExposomeMap-PM is designed as a modular model. It covers various particle types from multiple sources that exert effects on the respiratory, cardiovascular and neurological systems through different mechanisms. This design allows easy updates and extensions with new information, as discussed in previous publications (Mazein et al., 2023, 2021). The chosen scope provides a unifying depiction of interactions and data visualization in a single diagram, while the modular architecture enables targeted refinement without restructuring the resource as a whole. Expansions can include cell- and system-specific mechanisms, for example alveolar-capillary translocation, blood-brain barrier crossing or the olfactory route.

The ExposomeMap-PM resource can be used as a curated machine-readable basis that can be developed further into a computational model, while including particle concentration data and real-world exposure-effect relationships, showing the role of dose–response and temporal aspects. With additional refining to make it computationally executable, the resource offers potential for simulation of dynamic exposure-driven perturbations and identification of key regulatory nodes affected by fine and ultrafine particles. By capturing how molecular pathways respond to varying exposure levels and durations, the framework could support hypothesis generation and predictive assessment of exposure-related health outcomes.

Several directions are anticipated for the development and use of ExposomeMap-PM. A distinctive feature of the map is that it represents mechanisms for multiple particle types within a single connected structure. This design makes it possible to move beyond single-exposure type and trace how several pollutants acting together are linked to a common phenotype. On the Atlas of Cancer Signalling Network, structural analysis of this kind predicted a non-obvious combination of perturbations driving disease progression, which was subsequently confirmed *in vivo* (Chanrion et al., 2014; Jdey et al., 2017). Converting the core blocks into an executable logical model would extend this further. It would allow combined exposure-response behaviour to be simulated under varying dose and duration, and candidate interventions could be ranked by their predicted effect as demonstrated for disease maps parameterised with patient cohort data (Hemedan et al., 2024).

The associations to specific diseases within the ExposomeMap-PM depend on exposure concentration, exposure time, age, sex, genetics, existing health conditions and co-exposures including smoking. Real-world exposure is also a mixture and not a single type of particle. For example, volcanic and desert dust can carry silica minerals, transition metals, secondary inorganic and organic elements. This way, the observed effects are a combination of factors and co-occurring components. The contribution of the ExposomeMap-PM is not to imply direct causation but to present the shared molecular mechanisms linked to particle exposure to disease-inducing mechanisms, as well as to point to shared mechanisms for different types of particles. The ExposomeMap places these mechanisms from many sources within one shared space, shows overlaps and coverage, and links them to adverse outcomes and related diseases. This framing helps to explain complex dependencies on particle reactivity and exposure context in contrast to only taking into account concentration and exposure time.

ExposomeMap-PM relates to several existing resources and prior works. The map was developed using the established AOP-to-map workflow (Mazein et al., 2025, 2024), previously applied to immune-related adverse outcome pathways and here tailored to particle-induced toxicity. In particular, the focus is on untangling how particle source and particle type connect through receptor engagement and downstream signalling to respiratory, cardiovascular and neurological outcomes. Published particle AOP networks from the literature (Ding et al., 2024; Halappanavar et al., 2020; Li T et al., 2022; Yu et al., 2024; Zhang et al., 2024) were used to plan the scope of the map (Figure 2). These networks operate at the level of key-event connections, and ExposomeMap-PM moves the representation to the molecular level and supports the overlay of omics data. In addition to literature-based map development, Reactome (https://reactome.org/) and KEGG (https://www.genome.jp/kegg/) were used where relevant during map construction and cross-checking: detoxification of reactive oxygen species (Reactome:R-HSA-3299685) and reactive oxygen species (KEGG:hsa05208), as marked on the map. For future refinement and extensions, in addition to direct literature search, AOP-Wiki (https://aopwiki.org/) and the Comparative Toxicogenomics Database (CTD, https://ctdbase.org/) can provide complementary resources for planning and identifying relevant mechanisms, and WikiPathways (https://www.wikipathways.org/), Reactome and KEGG can be reused for molecular-level details.

The ExposomeMap-PM effort aligns with the 3R principle (Replacement, Reduction, Refinement) in animal research: it organises available knowledge into a structured resource that can contribute to reducing the need for animal studies. Replacement (direct alignment): by presenting an AOP-based molecular-level map, the resource supports interpretation of omics-based New Approach Methodologies (NAMs) data, so research questions can be addressed without animal experiments. Reduction (direct alignment): by reviewing and identifying which processes and endpoints are relevant, the map allows studies to be planned selectively, reusing existing published evidence to minimise redundant research and narrow the focus, this way lowering the number of animals needed for verification experiments. Refinement (indirect alignment): by clarifying which measured molecules and parameters are most informative, the map can guide the design of hypothesis verification studies that still require animals. The mechanistic molecular-level, and cellular-level maps built on the basis of the AOP concept, are critical in advancing NAMs for reducing reliance on animal testing (https://www.epa.gov/chemical-research/adverse-outcome-pathways, https://www.pharmtech.com/view/ema-encourages-use-of-new-approach-methodologies-to-reduce-animal-testing) (Burden et al., 2015).

The resource is designed for integration into exposomic data platforms such as EXPOsignalZ (https://www.exposignalz.com) and ENVESOME (https://envesome.eu), and connects the MARKOPOLO project with other EU efforts within and outside of the ExpoHealthNet Cluster (https://expohealthnet.eu), a coordination initiative that brings together projects addressing exposome research and the environmental determinants of health. This alignment provides a framework for connecting the molecular mechanisms underlying the toxicity of fine- and ultrafine-particles to real-world exposomic datasets. It is intended to enable predictive toxicology and personalised exposure-response modelling and supports the identification of vulnerable populations and mechanistic biomarkers for risk assessment.

## Conclusion

The ExposomeMap-PM provides a mechanistic layer of understanding between epidemiological evidence of particle-induced harmful effects and the molecular events that drive them and connect to outcomes. This is a reusable and extendable resource that provides a shared understanding of relevant mechanisms. By advancing the physiology-level adverse outcome pathway view into a molecular-level map, the resource makes it possible to trace how receptor activation, phagocytic uptake, ion channel disruption and surface-driven oxidative chemistry go through shared signalling cascades to downstream outcomes. The transcriptomics and proteomics data overlay demonstrates how condition-specific molecular signatures can be projected and interpreted in the context of these mechanisms. The current version focuses on first-wave cellular responses within a generalised cellular context. The modular structure enables further integration of cell-type-specific second-wave mechanisms and feedback loops as the resource develops and offers a basis for computational modelling.

## Data Availability

The maps are freely accessible online via https://disease-maps.io/markopolo/ under the Creative Commons Attribution 4.0 International License (CC BY 4.0). The archival snapshot is deposited on Zenodo at https://doi.org/10.5281/zenodo.22162142 and includes the CellDesigner XML current project file, source file, SBGN-ML export and overlay files.
