# Supplementary material for: ExposomeMap-PM: fine and ultrafine particle-induced toxicity - from adverse outcome pathways to systems biomedicine disease models
Source: Front Toxicol. 2026 Oct 27;8:1951777. doi: 10.3389/ftox.2026.1951777 (PMC13749717; doi:10.3389/ftox.2026.1951777)
Supplement: Supplementary file 1 [file DataSheet1.pdf]

Supplementary Table S1. Combining the system-specific AOP diagrams of Li et al. (2022) into the integrated AOP network. The first column lists the source key events with the source diagram marked RE, respiratory; CV, cardiovascular; NE, neurological. MIE, molecular initiating event; KE, key event; AO, adverse outcome. Only the nodes that changed during integration are shown; key events transferred unchanged are not listed, for example KE 1998 Microglia activation (NE). Terms in the comment column: **merged**, one key event present in more than one diagram combined into a single node; **absorbed**, a key event combined into a broader node; **generalised**, a tissue-specific label replaced by a broader one. Where the source diagrams labelled the same key event differently, a single consistent class was used in the integrated network.

**Source key events**	**Integrated node**	**Comment**
KE 151 Inflammatory pathways activation (RE, CV, NE)	KE 151 Inflammatory pathway activation	Merged
KE 149 Inflammatory response (RE, CV, NE); KE 1497 Infiltration of inflammatory cells (RE); KE 1198 Alveolar macrophages activation (RE)	KE 149 Inflammatory response	KE 149 merged across the three diagrams; KE 1497 and KE 1198 absorbed into it
KE 1496 Secretion of inflammatory factors (RE, CV, NE)	KE 1496 Secretion of inflammatory factors	Merged
KE 1115 ROS generation (RE, CV, NE)	KE 1115 ROS generation	Merged
KE 1392 Oxidative stress (RE, CV, NE)	KE 1392 Oxidative stress	Merged
KE 1445 Lipid peroxidation (RE, CV)	KE 1445 Lipid peroxidation	Merged
KE 1239 Altered gene expression (RE, CV, NE); KE 1194 DNA damage (RE); KE 165 AhR driven gene expression changes (RE)	KE 1239 Altered gene expression	KE 1239 merged across the three diagrams; the source labelled it as MIE in the respiratory figure and KE in the cardiovascular and neurological figures; a single class was used in the integrated network; KE 1194 and KE 165 absorbed, their downstream link to apoptosis re-routed through this node
KE 1994 Ferroptosis (RE, CV)	KE 1994 Ferroptosis	Merged
KE 1262 Apoptosis (RE); KE 1918 Myocardial apoptosis (CV)	KE 1262 Apoptosis	Merged into one node under the generic label "Apoptosis"; tissue-specific KE 1918 generalised
KE 1945 Dysfunctional autophagy (RE); KE 1927 Vascular endothelial cell autophagy (CV)	KE 1945 Autophagy dysfunction	Merged into one node harmonised to "Autophagy dysfunction"; tissue-specific KE 1927 generalised
