# Supplementary material for: ExposomeMap-PM: fine and ultrafine particle-induced toxicity - from adverse outcome pathways to systems biomedicine disease models
Source: Front Toxicol. 2026 Oct 27;8:1951777. doi: 10.3389/ftox.2026.1951777 (PMC13749717; doi:10.3389/ftox.2026.1951777)
Supplement: Supplementary file 2 [file DataSheet2.pdf]

Supplementary File S2. Reference library

1. Abdillah, S.F.I., Wang, Y.-F., 2023. Ambient ultrafine particle (PM0.1): Sources, characteristics, measurements and exposure implications on human health. Environ Res 218, 115061. https://doi.org/10.1016/j.envres.2022.115061
2. Afthab, M., Hambo, S., Kim, H., Alhamad, A., Harb, H., 2024. Particulate matter-induced epigenetic modifications and lung complications. European Respiratory Review 33. https://doi.org/10.1183/16000617.0129-2024
3. Ahmad, S., K G, N., Mani Babu, A., Ranjan, R., Kumar, P., 2024. Association Between Ambient Air Pollution and Attention-Deficit/Hyperactivity Disorder (ADHD) in Children: A Systematic Review and Meta-Analysis. Cureus 16, e71527. https://doi.org/10.7759/cureus.71527
4. Aluri, J., Cooper, M.A., Schuettpelz, L.G., 2021. Toll-Like Receptor Signaling in the Establishment and Function of the Immune System. Cells 10, 1374. https://doi.org/10.3390/cells10061374
5. An, N., Gao, Y., Si, Z., Zhang, H., Wang, L., Tian, C., Yuan, M., Yang, X., Li, X., Shang, H., Xiong, X., Xing, Y., 2019. Regulatory Mechanisms of the NLRP3 Inflammasome, a Novel Immune-Inflammatory Marker in Cardiovascular Diseases. Front Immunol 10, 1592. https://doi.org/10.3389/fimmu.2019.01592
6. Andrews, J.P.M., Joshi, S.S., Tzolos, E., Syed, M.B., Cuthbert, H., Crica, L.E., Lozano, N., Okwelogu, E., Raftis, J.B., Bruce, L., Poland, C.A., Duffin, R., Fokkens, P.H.B., Boere, A.J.F., Leseman, D.L.A.C., Megson, I.L., Whitfield, P.D., Ziegler, K., Tammireddy, S., Hadjidemetriou, M., Bussy, C., Cassee, F.R., Newby, D.E., Kostarelos, K., Miller, M.R., 2024. First-in-human controlled inhalation of thin graphene oxide nanosheets to study acute cardiorespiratory responses. Nat Nanotechnol 19, 705–714. https://doi.org/10.1038/s41565-023-01572-3
7. Angel, S., Eades, L.J., Sim, G., Czopek, A., Dhaun, N., Krystek, P., Miller, M.R., 2024. New insights into the association of air pollution and kidney diseases by tracing gold nanoparticles with inductively coupled plasma mass spectrometry. Anal Bioanal Chem 416, 2683–2689. https://doi.org/10.1007/s00216-023-05105-8
8. Araujo, J.A., Barajas, B., Kleinman, M., Wang, X., Bennett, B.J., Gong, K.W., Navab, M., Harkema, J., Sioutas, C., Lusis, A.J., Nel, A.E., 2008. Ambient particulate pollutants in the ultrafine range promote early atherosclerosis and systemic oxidative stress. Circ Res 102, 589–596. https://doi.org/10.1161/CIRCRESAHA.107.164970
9. Becker, S., Dailey, L., Soukup, J.M., Silbajoris, R., Devlin, R.B., 2005. TLR-2 is involved in airway epithelial cell response to air pollution particles. Toxicology and Applied Pharmacology 203, 45–52. https://doi.org/10.1016/j.taap.2004.07.007
10. Bevan, G.H., Al-Kindi, S.G., Brook, R., Rajagopalan, S., 2021. Ambient Air Pollution and Atherosclerosis: Recent Updates. Curr Atheroscler Rep 23, 63. https://doi.org/10.1007/s11883-021-00958-9
11. Bowe, B., Xie, Y., Li, T., Yan, Y., Xian, H., Al-Aly, Z., 2018. Particulate Matter Air Pollution and the Risk of Incident CKD and Progression to ESRD. J Am Soc Nephrol 29, 218–230. https://doi.org/10.1681/ASN.2017030253
12. Brook, R.D., 2008. Cardiovascular effects of air pollution. Clin Sci (Lond) 115, 175–187. https://doi.org/10.1042/CS20070444
13. Brugge, D., Li, J., Zamore, W., 2023. On the Need for Human Studies of PM Exposure Activation of the NLRP3 Inflammasome. Toxics 11, 202. https://doi.org/10.3390/toxics11030202
14. Cachon, B.F., Firmin, S., Verdin, A., Ayi-Fanou, L., Billet, S., Cazier, F., Martin, P.J., Aissi, F., Courcot, D., Sanni, A., Shirali, P., 2014. Proinflammatory effects and oxidative stress within human bronchial epithelial cells exposed to atmospheric particulate matter (PM(2.5) and

PM(>2.5)) collected from Cotonou, Benin. Environ Pollut 185, 340–351. https://doi.org/10.1016/j.envpol.2013.10.026
15. Calderón-Garcidueñas, L., Torres-Jardón, R., Franco-Lira, M., Kulesza, R., González-Maciel, A., Reynoso-Robles, R., Brito-Aguilar, R., García-Arreola, B., Revueltas-Ficachi, P., Barrera-Velázquez, J.A., García-Alonso, G., García-Rojas, E., Mukherjee, P.S., Delgado-Chávez, R., 2020. Environmental Nanoparticles, SARS-CoV-2 Brain Involvement, and Potential Acceleration of Alzheimer's and Parkinson's Diseases in Young Urbanites Exposed to Air Pollution. Journal of Alzheimer's Disease 78, 479–503. https://doi.org/10.3233/JAD-200891
16. Chan, J.Y.W., Tsui, J.C.C., Law, P.T.W., So, W.K.W., Leung, D.Y.P., Sham, M.M.K., Tsui, S.K.W., Chan, C.W.H., 2018. Regulation of TLR4 in silica-induced inflammation: An underlying mechanism of silicosis. International Journal of Medical Sciences 15, 986–991. https://doi.org/10.7150/ijms.24715
17. Chan, Y.-C., Huang, Y.-W., Chuang, S.-H., Kuo, Y.-J., Chen, Y.-P., 2025. A decade of exposure: Long-term air pollution and its impact on osteoarthritis: A nationwide cohort study in Taiwan. Osteoarthritis and Cartilage 0. https://doi.org/10.1016/j.joca.2025.04.011
18. Chaudhuri, N., Paiva, C., Donaldson, K., Duffin, R., Parker, L.C., Sabroe, I., 2010. Diesel exhaust particles override natural injury-limiting pathways in the lung. American Journal of Physiology-Lung Cellular and Molecular Physiology 299, L263–L271. https://doi.org/10.1152/ajplung.00297.2009
19. Chauhan, B.V.S., Corada, K., Young, C., Smallbone, K.L., Wyche, K.P., 2024. Review on Sampling Methods and Health Impacts of Fine (PM2.5, ≤2.5 µm) and Ultrafine (UFP, PM0.1, ≤0.1 µm) Particles. Atmosphere 15, 572. https://doi.org/10.3390/atmos15050572
20. Chen, X., Liu, J., Zhou, J., Wang, J., Chen, C., Song, Y., Pan, J., 2018. Urban particulate matter (PM) suppresses airway antibacterial defence. Respir Res 19, 5. https://doi.org/10.1186/s12931-017-0700-0
21. Chen, Y., Wu, Y., Qi, Y., Liu, S., 2023. Cell Death Pathways: The Variable Mechanisms Underlying Fine Particulate Matter-Induced Cytotoxicity. ACS Nanosci. Au 3, 130–139. https://doi.org/10.1021/acsnanoscienceau.2c00059
22. Chen, Z., Liu, P., Xia, X., Wang, L., Li, X., 2022. The underlying mechanism of PM2.5-induced ischemic stroke. Environ Pollut 310, 119827. https://doi.org/10.1016/j.envpol.2022.119827
23. Cochard, M., Ledoux, F., Landkocz, Y., 2020. Atmospheric fine particulate matter and epithelial mesenchymal transition in pulmonary cells: state of the art and critical review of the in vitro studies. Journal of Toxicology and Environmental Health, Part B 23, 293–318. https://doi.org/10.1080/10937404.2020.1816238
24. Delhez, M.-L., Bosmans, M., Rodriguez, L.R., Gillard, A., Blacher, S., Blomme, A., Close, P., Machiels, B., Nokin, M.-J., Cataldo, D., 2026. Exposure to diesel particulates induces an immunosuppressive microenvironment that promotes the progression of lung cancer. Neoplasia 71, 101255. https://doi.org/10.1016/j.neo.2025.101255
25. Deng, Q., Deng, L., Miao, Y., Guo, X., Li, Y., 2019. Particle deposition in the human lung: Health implications of particulate matter from different sources. Environ Res 169, 237–245. https://doi.org/10.1016/j.envres.2018.11.014
26. Dovrou, E., Lelieveld, S., Mishra, A., Pöschl, U., Berkemeier, T., 2023. Influence of ambient and endogenous H 2 O 2 on reactive oxygen species concentrations and OH radical production in the respiratory tract. Environmental Science: Atmospheres 3, 1066–1074. https://doi.org/10.1039/D2EA00179A
27. Du, X., Guan, L., Chen, C., Wang, X., Geng, X., 2025. Long-term exposure to PM2.5 exacerbates dopaminergic neuronal loss through CpG hypermethylation induced down-regulation of PINK1 and DJ-1 genes. Sci Rep 15, 10778. https://doi.org/10.1038/s41598-025-89422-1
28. Dutheil, F., Comptour, A., Morlon, R., Mermillod, M., Pereira, B., Baker, J.S., Charkhabi, M., Clinchamps, M., Bourdel, N., 2021. Autism spectrum disorder and air pollution: A systematic

review and meta-analysis. Environ Pollut 278, 116856. https://doi.org/10.1016/j.envpol.2021.116856
29. Dvonch, J.T., Brook, R.D., Keeler, G.J., Rajagopalan, S., D'Alecy, L.G., Marsik, F.J., Morishita, M., Yip, F.Y., Brook, J.R., Timm, E.J., Wagner, J.G., Harkema, J.R., 2004. Effects of concentrated fine ambient particles on rat plasma levels of asymmetric dimethylarginine. Inhal Toxicol 16, 473–480. https://doi.org/10.1080/08958370490439678
30. El-Zayat, S.R., Sibaii, H., Mannaa, F.A., 2019. Toll-like receptors activation, signaling, and targeting: an overview. Bulletin of the National Research Centre 43, 187. https://doi.org/10.1186/s42269-019-0227-2
31. Eltom, S., Belvisi, M.G., Stevenson, C.S., Maher, S.A., Dubuis, E., Fitzgerald, K.A., Birrell, M.A., 2014. Role of the Inflammasome-Caspase1/11-IL-1/18 Axis in Cigarette Smoke Driven Airway Inflammation: An Insight into the Pathogenesis of COPD. PLOS ONE 9, e112829. https://doi.org/10.1371/journal.pone.0112829
32. Fathieh, S., Grieve, S.M., Negishi, K., Figtree, G.A., 2023. Potential Biological Mediators of Myocardial and Vascular Complications of Air Pollution—A State-of-the-Art Review. Heart, Lung and Circulation 32, 26–42. https://doi.org/10.1016/j.hlc.2022.11.014
33. Favor, O.K., Pestka, J.J., Bates, M.A., Lee, K.S.S., 2021. Centrality of Myeloid-Lineage Phagocytes in Particle-Triggered Inflammation and Autoimmunity. Front. Toxicol. 3. https://doi.org/10.3389/ftox.2021.777768
34. Fiordelisi, A., Piscitelli, P., Trimarco, B., Coscioni, E., Iaccarino, G., Sorriento, D., 2017. The mechanisms of air pollution and particulate matter in cardiovascular diseases. Heart Fail Rev 22, 337–347. https://doi.org/10.1007/s10741-017-9606-7
35. Flood-Garibay, J.A., Angulo-Molina, A., Méndez-Rojas, M.Á., 2023. Particulate matter and ultrafine particles in urban air pollution and their effect on the nervous system. Environ. Sci.: Processes Impacts 25, 704–726. https://doi.org/10.1039/D2EM00276K
36. Fonken, L.K., Xu, X., Weil, Z.M., Chen, G., Sun, Q., Rajagopalan, S., Nelson, R.J., 2011. Air pollution impairs cognition, provokes depressive-like behaviors and alters hippocampal cytokine expression and morphology. Mol Psychiatry 16, 987–995, 973. https://doi.org/10.1038/mp.2011.76
37. Fortoul, T.I., Rojas-Lemus, M., Rodriguez-Lara, V., Cano-Gutierrez, G., Gonzalez-Villalva, A., Ustarroz-Cano, M., Garcia-Pelaez, I., Lopez-Valdez, N., Falcon-Rodriguez, C.I., Silva-Martinez, J., Gonzalez-Rendon, E.S., Montaño, L.F., Cano-Gutierrez, B., Bizarro-Nevares, P., Colin-Barenque, L., 2011. Air Pollution and Its Effects in the Respiratory System, in: The Impact of Air Pollution on Health, Economy, Environment and Agricultural Sources. IntechOpen. https://doi.org/10.5772/17766
38. Frias, D.P., Gomes, R.L.N., Yoshizaki, K., Carvalho-Oliveira, R., Matsuda, M., Junqueira, M. de S., Teodoro, W.R., Vasconcellos, P. de C., Pereira, D.C. de A., Conceição, P.R. da, Saldiva, P.H.N., Mauad, T., Macchione, M., 2020. Nrf2 positively regulates autophagy antioxidant response in human bronchial epithelial cells exposed to diesel exhaust particles. Sci Rep 10, 3704. https://doi.org/10.1038/s41598-020-59930-3
39. Fuller, R., Landrigan, P.J., Balakrishnan, K., Bathan, G., Bose-O'Reilly, S., Brauer, M., Caravanos, J., Chiles, T., Cohen, A., Corra, L., Cropper, M., Ferraro, G., Hanna, J., Hanrahan, D., Hu, H., Hunter, D., Janata, G., Kupka, R., Lanphear, B., Lichtveld, M., Martin, K., Mustapha, A., Sanchez-Triana, E., Sandilya, K., Schaefli, L., Shaw, J., Seddon, J., Suk, W., Téllez-Rojo, M.M., Yan, C., 2022. Pollution and health: a progress update. The Lancet Planetary Health 6, e535–e547. https://doi.org/10.1016/S2542-5196(22)00090-0
40. Gavito-Covarrubias, D., Ramírez-Díaz, I., Guzmán-Linares, J., Limón, I.D., Manuel-Sánchez, D.M., Molina-Herrera, A., Coral-García, M.Á., Anastasio, E., Anaya-Hernández, A., López-Salazar, P., Juárez-Díaz, G., Martínez-Juárez, J., Torres-Jácome, J., Albarado-Ibáñez, A., Martínez-Laguna, Y., Morán, C., Rubio, K., 2024. Epigenetic mechanisms of particulate

matter exposure: air pollution and hazards on human health. Front. Genet. 14. https://doi.org/10.3389/fgene.2023.1306600
41. GBD 2021 Risk Factors Collaborators, 2024. Global burden and strength of evidence for 88 risk factors in 204 countries and 811 subnational locations, 1990-2021: a systematic analysis for the Global Burden of Disease Study 2021. Lancet 403, 2162–2203. https://doi.org/10.1016/S0140-6736(24)00933-4
42. GBD 2023 Disease and Injury and Risk Factor Collaborators, 2025. Burden of 375 diseases and injuries, risk-attributable burden of 88 risk factors, and healthy life expectancy in 204 countries and territories, including 660 subnational locations, 1990-2023: a systematic analysis for the Global Burden of Disease Study 2023. Lancet 406, 1873–1922. https://doi.org/10.1016/S0140-6736(25)01637-X
43. Geiser, M., Rothen-Rutishauser, B., Kapp, N., Schürch, S., Kreyling, W., Schulz, H., Semmler, M., Im Hof, V., Heyder, J., Gehr, P., 2005. Ultrafine particles cross cellular membranes by nonphagocytic mechanisms in lungs and in cultured cells. Environ Health Perspect 113, 1555–1560. https://doi.org/10.1289/ehp.8006
44. Gong, F.-H., Liu, L., Wang, X., Xiang, Q., Yi, X., Jiang, D.-S., 2025. Ferroptosis induced by environmental pollutants and its health implications. Cell Death Discov. 11, 20. https://doi.org/10.1038/s41420-025-02305-2
45. González-Maciel, A., Reynoso-Robles, R., Torres-Jardón, R., Mukherjee, P.S., Calderón-Garcidueñas, L., 2017. Combustion-Derived Nanoparticles in Key Brain Target Cells and Organelles in Young Urbanites: Culprit Hidden in Plain Sight in Alzheimer's Disease Development. J Alzheimers Dis 59, 189–208. https://doi.org/10.3233/JAD-170012
46. Han, D., Chen, R., Kan, H., Xu, Y., 2023. The bio-distribution, clearance pathways, and toxicity mechanisms of ambient ultrafine particles. Eco-Environment & Health 2, 95–106. https://doi.org/10.1016/j.eehl.2023.06.001
47. Hazari, M.S., Haykal-Coates, N., Winsett, D.W., Krantz, Q.T., King, C., Costa, D.L., Farraj, A.K., 2011. TRPA1 and sympathetic activation contribute to increased risk of triggered cardiac arrhythmias in hypertensive rats exposed to diesel exhaust. Environ Health Perspect 119, 951–957. https://doi.org/10.1289/ehp.1003200
48. Hill, W., Lim, E.L., Weeden, C.E., Lee, C., Augustine, M., Chen, K., Kuan, F.-C., Marongiu, F., Evans, E.J., Moore, D.A., Rodrigues, F.S., Pich, O., Bakker, B., Cha, H., Myers, R., van Maldegem, F., Boumelha, J., Veeriah, S., Rowan, A., Naceur-Lombardelli, C., Karasaki, T., Sivakumar, M., De, S., Caswell, D.R., Nagano, A., Black, J.R.M., Martínez-Ruiz, C., Ryu, M.H., Huff, R.D., Li, S., Favé, M.-J., Magness, A., Suárez-Bonnet, A., Priestnall, S.L., Lüchtenborg, M., Lavelle, K., Pethick, J., Hardy, S., McRonald, F.E., Lin, M.-H., Troccoli, C.I., Ghosh, M., Miller, Y.E., Merrick, D.T., Keith, R.L., Al Bakir, M., Bailey, C., Hill, M.S., Saal, L.H., Chen, Y., George, A.M., Abbosh, C., Kanu, N., Lee, S.-H., McGranahan, N., Berg, C.D., Sasieni, P., Houlston, R., Turnbull, C., Lam, S., Awadalla, P., Grönroos, E., Downward, J., Jacks, T., Carlsten, C., Malanchi, I., Hackshaw, A., Litchfield, K., TRACERx Consortium, DeGregori, J., Jamal-Hanjani, M., Swanton, C., 2023. Lung adenocarcinoma promotion by air pollutants. Nature 616, 159–167. https://doi.org/10.1038/s41586-023-05874-3
49. Hogan, M.K., Kovalycsik, T., Sun, Q., Rajagopalan, S., Nelson, R.J., 2015. Combined effects of exposure to dim light at night and fine particulate matter on C3H/HeNHsd mice. Behav Brain Res 294, 81–88. https://doi.org/10.1016/j.bbr.2015.07.033
50. Holme, J.A., Vondráček, J., Machala, M., Lagadic-Gossmann, D., Vogel, C.F.A., Le Ferrec, E., Sparfel, L., Øvrevik, J., 2023. Lung cancer associated with combustion particles and fine particulate matter (PM2.5) - The roles of polycyclic aromatic hydrocarbons (PAHs) and the aryl hydrocarbon receptor (AhR). Biochemical Pharmacology 216, 115801. https://doi.org/10.1016/j.bcp.2023.115801
51. Hornung, V., Bauernfeind, F., Halle, A., Samstad, E.O., Kono, H., Rock, K.L., Fitzgerald, K.A., Latz, E., 2008. Silica crystals and aluminum salts activate the NALP3 inflammasome

through phagosomal destabilization. Nat Immunol 9, 847–856. https://doi.org/10.1038/ni.1631
52. Hsu, Y.-H., Chuang, H.-C., Lee, Y.-H., Lin, Y.-F., Chen, Y.-J., Hsiao, T.-C., Wu, M.-Y., Chiu, H.-W., 2019. Traffic-related particulate matter exposure induces nephrotoxicity in vitro and in vivo. Free Radic Biol Med 135, 235–244. https://doi.org/10.1016/j.freeradbiomed.2019.03.008
53. Jin, Y., Zhu, M., Guo, Y., Foreman, D., Feng, F., Duan, G., Wu, W., Zhang, W., 2019. Fine particulate matter (PM2.5) enhances FcεRI-mediated signaling and mast cell function. Cellular Signalling 57, 102–109. https://doi.org/10.1016/j.cellsig.2019.01.010
54. Kampfrath, T., Maiseyeu, A., Ying, Z., Shah, Z., Deiuliis, J.A., Xu, X., Kherada, N., Brook, R.D., Reddy, K.M., Padture, N.P., Parthasarathy, S., Chen, L.C., Moffatt-Bruce, S., Sun, Q., Morawietz, H., Rajagopalan, S., 2011. Chronic fine particulate matter exposure induces systemic vascular dysfunction via NADPH oxidase and TLR4 pathways. Circ Res 108, 716–726. https://doi.org/10.1161/CIRCRESAHA.110.237560
55. Kaur, M., Chandel, J., Malik, J., Naura, A.S., 2022. Particulate matter in COPD pathogenesis: an overview. Inflamm. Res. 71, 797–815. https://doi.org/10.1007/s00011-022-01594-y
56. Kayalar, Ö., Rajabi, H., Konyalilar, N., Mortazavi, D., Aksoy, G.T., Wang, J., Bayram, H., 2024. Impact of particulate air pollution on airway injury and epithelial plasticity; underlying mechanisms. Front. Immunol. 15. https://doi.org/10.3389/fimmu.2024.1324552
57. Kelly, F.J., Fussell, J.C., 2012. Size, source and chemical composition as determinants of toxicity attributable to ambient particulate matter. Atmospheric Environment 60, 504–526. https://doi.org/10.1016/j.atmosenv.2012.06.039
58. Kim, H.S., Na, H.-W., Jang, Y., Kim, S.J., Kee, N.G., Shin, D.Y., Choi, H., Kim, H.-J., Seo, Y.R., 2022. Integrative analysis to explore the biological association between environmental skin diseases and ambient particulate matter. Sci Rep 12, 9750. https://doi.org/10.1038/s41598-022-13001-x
59. Kim, J., Natarajan, S., Vaickus, L.J., Bouchard, J.C., Beal, D., Cruikshank, W.W., Remick, D.G., 2011. Diesel exhaust particulates exacerbate asthma-like inflammation by increasing CXC chemokines. Am J Pathol 179, 2730–2739. https://doi.org/10.1016/j.ajpath.2011.08.008
60. Kim, S.Y., Kim, J.K., Park, S.H., Kim, B.-G., Jang, A.-S., Oh, S.H., Lee, J.H., Suh, M.-W., Park, M.K., 2018. Effects of inhaled particulate matter on the central nervous system in mice. Neurotoxicology 67, 169–177. https://doi.org/10.1016/j.neuro.2018.06.001
61. Kuntic, M., Kuntic, I., Cleppien, D., Pozzer, A., Nußbaum, D., Oelze, M., Junglas, T., Strohm, L., Ubbens, H., Daub, S., Bayo Jimenez, M.T., Danckwardt, S., Berkemeier, T., Hahad, O., Kohl, M., Steven, S., Stroh, A., Lelieveld, J., Münzel, T., Daiber, A., 2025. Differential inflammation, oxidative stress and cardiovascular damage markers of nano- and micro-particle exposure in mice: Implications for human disease burden. Redox Biol 83, 103644. https://doi.org/10.1016/j.redox.2025.103644
62. Kuntic, M., Kuntic, I., Krishnankutty, R., Gericke, A., Oelze, M., Junglas, T., Bayo Jimenez, M.T., Stamm, P., Nandudu, M., Hahad, O., Keppeler, K., Daub, S., Vujacic-Mirski, K., Rajlic, S., Strohm, L., Ubbens, H., Tang, Q., Jiang, S., Ruan, Y., Macleod, K.G., Steven, S., Berkemeier, T., Pöschl, U., Lelieveld, J., Kleinert, H., von Kriegsheim, A., Daiber, A., Münzel, T., 2023. Co-exposure to urban particulate matter and aircraft noise adversely impacts the cerebro-pulmonary-cardiovascular axis in mice. Redox Biology 59, 102580. https://doi.org/10.1016/j.redox.2022.102580
63. Laing, S., Wang, G., Briazova, T., Zhang, C., Wang, A., Zheng, Z., Gow, A., Chen, A.F., Rajagopalan, S., Chen, L.C., Sun, Q., Zhang, K., 2010. Airborne particulate matter selectively activates endoplasmic reticulum stress response in the lung and liver tissues. Am J Physiol Cell Physiol 299, C736-749. https://doi.org/10.1152/ajpcell.00529.2009
64. Law, C.W., Chen, Y., Shi, W., Smyth, G.K., 2014. voom: Precision weights unlock linear model analysis tools for RNA-seq read counts. Genome Biol 15, R29. https://doi.org/10.1186/gb-2014-15-2-r29

65. Lederer, A.M., Fredriksen, P.M., Nkeh-Chungag, B.N., Everson, F., Strijdom, H., De Boever, P., Goswami, N., 2021. Cardiovascular effects of air pollution: current evidence from animal and human studies. Am J Physiol Heart Circ Physiol 320, H1417–H1439. https://doi.org/10.1152/ajpheart.00706.2020
66. Lee, J., Weerasinghe-Mudiyanselage, P.D.E., Kim, B., Kang, S., Kim, J.-S., Moon, C., 2024. Impact of diesel particulate matter on the olfactory bulb of mice: insights from behavioral, histological, and molecular assessments. Mol. Cell. Toxicol. 20, 735–745. https://doi.org/10.1007/s13273-023-00414-6
67. Leikauf, G.D., Kim, S.-H., Jang, A.-S., 2020. Mechanisms of ultrafine particle-induced respiratory health effects. Exp Mol Med 52, 329–337. https://doi.org/10.1038/s12276-020-0394-0
68. Lelieveld, J., Haines, A., Burnett, R., Tonne, C., Klingmüller, K., Münzel, T., Pozzer, A., 2023. Air pollution deaths attributable to fossil fuels: observational and modelling study. BMJ 383, e077784. https://doi.org/10.1136/bmj-2023-077784
69. Lelieveld, S., Wilson, J., Dovrou, E., Mishra, A., Lakey, P.S.J., Shiraiwa, M., Pöschl, U., Berkemeier, T., 2021. Hydroxyl Radical Production by Air Pollutants in Epithelial Lining Fluid Governed by Interconversion and Scavenging of Reactive Oxygen Species. Environ. Sci. Technol. 55, 14069–14079. https://doi.org/10.1021/acs.est.1c03875
70. Li, H., Cai, J., Chen, R., Zhao, Z., Ying, Z., Wang, L., Chen, J., Hao, K., Kinney, P.L., Chen, H., Kan, H., 2017. Particulate Matter Exposure and Stress Hormone Levels: A Randomized, Double-Blind, Crossover Trial of Air Purification. Circulation 136, 618–627. https://doi.org/10.1161/CIRCULATIONAHA.116.026796
71. Li, Q.-Q., Guo, Y.-T., Yang, J.-Y., Liang, C.-S., 2023. Review on main sources and impacts of urban ultrafine particles: Traffic emissions, nucleation, and climate modulation. Atmospheric Environment: X 19, 100221. https://doi.org/10.1016/j.aeaoa.2023.100221
72. Li, T., Yu, Y., Sun, Z., Duan, J., 2022. A comprehensive understanding of ambient particulate matter and its components on the adverse health effects based from epidemiological and laboratory evidence. Particle and Fibre Toxicology 19, 67. https://doi.org/10.1186/s12989-022-00507-5
73. Li, W., Lin, G., Xiao, Z., Zhang, Y., Li, B., Zhou, Y., Ma, Y., Chai, E., 2022. A review of respirable fine particulate matter (PM2.5)-induced brain damage. Front Mol Neurosci 15, 967174. https://doi.org/10.3389/fnmol.2022.967174
74. Li, Xiaobo, Zhang, Y., Li, B., Yang, H., Cui, J., Li, Xiaoyan, Zhang, X., Sun, H., Meng, Q., Wu, S., Li, S., Wang, J., Aschner, M., Chen, R., 2020. Activation of NLRP3 in microglia exacerbates diesel exhaust particles-induced impairment in learning and memory in mice. Environment International 136, 105487. https://doi.org/10.1016/j.envint.2020.105487
75. Li, Y., Ouyang, Y., Jiao, J., Xu, Z., Zhang, L., 2021. Exposure to environmental black carbon exacerbates nasal epithelial inflammation via the reactive oxygen species (ROS)–nucleotide-binding, oligomerization domain–like receptor family, pyrin domain containing 3 (NLRP3)–caspase-1–interleukin 1β (IL-1β) pathway. International Forum of Allergy & Rhinology 11, 773–783. https://doi.org/10.1002/alr.22669
76. Liang, S., Zhang, J., Ning, R., Du, Z., Liu, J., Batibawa, J.W., Duan, J., Sun, Z., 2020. The critical role of endothelial function in fine particulate matter-induced atherosclerosis. Particle and Fibre Toxicology 17, 61. https://doi.org/10.1186/s12989-020-00391-x
77. Liao, X., Liu, Y., Zheng, J., Zhao, X., Cui, L., Hu, S., Xia, T., Si, S., 2022. Diverse Pathways of Engineered Nanoparticle-Induced NLRP3 Inflammasome Activation. Nanomaterials 12, 3908. https://doi.org/10.3390/nano12213908
78. Lim, E.Y., Kim, G.-D., 2024. Particulate Matter-Induced Emerging Health Effects Associated with Oxidative Stress and Inflammation. Antioxidants 13, 1256. https://doi.org/10.3390/antiox13101256

79. Lin, C.-H., Liu, W.-S., Wan, C., Wang, H.-H., 2025. Induction of GPX4-regulated ferroptotic stress promotes epithelial-to-mesenchymal transition in renal tubule cells induced by PM2.5. Toxicol Appl Pharmacol 495, 117184. https://doi.org/10.1016/j.taap.2024.117184
80. Lin, M., Ji, X., Lv, Y., Cui, D., Xie, J., 2023. The Roles of TRAF3 in Immune Responses. Disease Markers 2023, 7787803. https://doi.org/10.1155/2023/7787803
81. Lintusaari, H., Lepistö, T., Saarikoski, S., Salo, L., Silvonen, V., Barreira, L.M.F., Aurela, M., Hoivala, J., Markkula, L., Ondracek, J., Wahle, T., Vojtisek-Lom, M., Topinka, J., Schins, R.P.F., Jalava, P., Timonen, H., Kanninen, K.M., Rönkkö, T., 2025. Importance of sub-23 nm particles in traffic environments: Particle number emission factors and extrathoracic deposition doses. Environ Pollut 369, 125835. https://doi.org/10.1016/j.envpol.2025.125835
82. Liu, C., Meng, L., Gao, Y., Chen, J., Zhu, M., Xiong, M., Xiao, T., Gu, X., Liu, Chaoyang, Li, T., Zhang, Z., 2024. PM2.5 triggers tau aggregation in a mouse model of tauopathy. JCI Insight 9, e176703. https://doi.org/10.1172/jci.insight.176703
83. Liu, L., Urch, B., Szyszkowicz, M., Speck, M., Leingartner, K., Shutt, R., Pelletier, G., Gold, D.R., Scott, J.A., Brook, J.R., Thorne, P.S., Silverman, F.S., 2017. Influence of exposure to coarse, fine and ultrafine urban particulate matter and their biological constituents on neural biomarkers in a randomized controlled crossover study. Environ Int 101, 89–95. https://doi.org/10.1016/j.envint.2017.01.010
84. Liu, W., Xu, L., Liang, X., Liu, X., Zhao, Y., Ma, C., Gao, L., 2020. Tim-4 in Health and Disease: Friend or Foe? Front. Immunol. 11. https://doi.org/10.3389/fimmu.2020.00537
85. Liu, Y., Zhang, W., Wang, H., Liu, H., Yu, Q., Luo, X., Feng, X., Yang, P., 2024. Fine particulate matter potentiates Th17-cell pathogenicity in experimental autoimmune uveitis via ferroptosis. Ecotoxicology and Environmental Safety 284, 116979. https://doi.org/10.1016/j.ecoenv.2024.116979
86. Liu, Z., Liu, W., Wei, H., Ping, Y., Yu, Z., Dong, Z., Ren, J., Zhang, S., Liu, S., 2025. Elevated lactate production exacerbates PM2.5-induced pulmonary fibrosis by stabilizing TGF-β1. J Adv Res S2090-1232(25)00587–9. https://doi.org/10.1016/j.jare.2025.07.057
87. Long, Y.-M., Yang, X.-Z., Yang, Q.-Q., Clermont, A.C., Yin, Y.-G., Liu, G.-L., Hu, L.-G., Liu, Q., Zhou, Q.-F., Liu, Q.S., Ma, Q.-C., Liu, Y.-C., Cai, Y., 2020. PM2.5 induces vascular permeability increase through activating MAPK/ERK signaling pathway and ROS generation. Journal of Hazardous Materials 386, 121659. https://doi.org/10.1016/j.jhazmat.2019.121659
88. Lü, S., Zhang, R., Yao, Z., Yi, F., Ren, J., Wu, M., Feng, M., Wang, Q., 2012. Size distribution of chemical elements and their source apportionment in ambient coarse, fine, and ultrafine particles in Shanghai urban summer atmosphere. Journal of Environmental Sciences 24, 882–890. https://doi.org/10.1016/S1001-0742(11)60870-X
89. Mandaglio-Collados, D., López-Gálvez, R., Ruiz-Alcaraz, A.J., López-García, C., Roldán, V., Lip, G.Y.H., Marín, F., Rivera-Caravaca, J.M., 2023. Impact of particulate matter on the incidence of atrial fibrillation and the risk of adverse clinical outcomes: A review. Science of The Total Environment 880, 163352. https://doi.org/10.1016/j.scitotenv.2023.163352
90. Manzano-Covarrubias, A.L., Yan, H., Luu, M.D.A., Gadjdjoe, P.S., Dolga, A.M., Schmidt, M., 2023. Unravelling the signaling power of pollutants. Trends in Pharmacological Sciences 44, 917–933. https://doi.org/10.1016/j.tips.2023.09.002
91. Massey, N., Puttachary, S., Bhat, S.M., Kanthasamy, A.G., Charavaryamath, C., 2019. HMGB1-RAGE Signaling Plays a Role in Organic Dust-Induced Microglial Activation and Neuroinflammation. Toxicol Sci 169, 579–592. https://doi.org/10.1093/toxsci/kfz071
92. Mazein, A., Acencio, M.L., Balaur, I., Rougny, A., Welter, D., Niarakis, A., Ramirez Ardila, D., Dogrusoz, U., Gawron, P., Satagopam, V., Gu, W., Kremer, A., Schneider, R., Ostaszewski, M., 2023. A guide for developing comprehensive systems biology maps of disease mechanisms: planning, construction and maintenance. Front Bioinform 3, 1197310. https://doi.org/10.3389/fbinf.2023.1197310

93. McDuffie, E., Martin, R., Yin, H., Brauer, M., 2021. Global Burden of Disease from Major Air Pollution Sources (GBD MAPS): A Global Approach. Res Rep Health Eff Inst 2021, 210.
94. Milici, A., Talavera, K., 2021. TRP Channels as Cellular Targets of Particulate Matter. International Journal of Molecular Sciences 22, 2783. https://doi.org/10.3390/ijms22052783
95. Miller, M.R., 2020. Oxidative stress and the cardiovascular effects of air pollution. Free Radic Biol Med 151, 69–87. https://doi.org/10.1016/j.freeradbiomed.2020.01.004
96. Miller, M.R., Raftis, J.B., Langrish, J.P., McLean, S.G., Samutrtai, P., Connell, S.P., Wilson, S., Vesey, A.T., Fokkens, P.H.B., Boere, A.J.F., Krystek, P., Campbell, C.J., Hadoke, P.W.F., Donaldson, K., Cassee, F.R., Newby, D.E., Duffin, R., Mills, N.L., 2017. Inhaled Nanoparticles Accumulate at Sites of Vascular Disease. ACS Nano 11, 4542–4552. https://doi.org/10.1021/acsnano.6b08551
97. Mills, N.L., Donaldson, K., Hadoke, P.W., Boon, N.A., MacNee, W., Cassee, F.R., Sandström, T., Blomberg, A., Newby, D.E., 2009. Adverse cardiovascular effects of air pollution. Nat Clin Pract Cardiovasc Med 6, 36–44. https://doi.org/10.1038/ncpcardio1399
98. Mills, N.L., Robinson, S.D., Fokkens, P.H.B., Leseman, D.L.A.C., Miller, M.R., Anderson, D., Freney, E.J., Heal, M.R., Donovan, R.J., Blomberg, A., Sandström, T., MacNee, W., Boon, N.A., Donaldson, K., Newby, D.E., Cassee, F.R., 2008. Exposure to concentrated ambient particles does not affect vascular function in patients with coronary heart disease. Environ Health Perspect 116, 709–715. https://doi.org/10.1289/ehp.11016
99. Mills, N.L., Törnqvist, H., Gonzalez, M.C., Vink, E., Robinson, S.D., Söderberg, S., Boon, N.A., Donaldson, K., Sandström, T., Blomberg, A., Newby, D.E., 2007. Ischemic and thrombotic effects of dilute diesel-exhaust inhalation in men with coronary heart disease. N Engl J Med 357, 1075–1082. https://doi.org/10.1056/NEJMoa066314
100. Mills, N.L., Törnqvist, H., Robinson, S.D., Gonzalez, M., Darnley, K., MacNee, W., Boon, N.A., Donaldson, K., Blomberg, A., Sandstrom, T., Newby, D.E., 2005. Diesel exhaust inhalation causes vascular dysfunction and impaired endogenous fibrinolysis. Circulation 112, 3930–3936. https://doi.org/10.1161/CIRCULATIONAHA.105.588962
101. Mo, Y., Mo, L., Zhang, Yue, Zhang, Yuanbao, Yuan, J., Zhang, Q., 2023. High glucose enhances the activation of NLRP3 inflammasome by ambient fine particulate matter in alveolar macrophages. Part Fibre Toxicol 20, 41. https://doi.org/10.1186/s12989-023-00552-8
102. Mohammadi, M.J., Zarea, K., Hatamzadeh, N., Salahshouri, A., Sharhani, A., 2022. Toxic Air Pollutants and Their Effect on Multiple Sclerosis: A Review Study. Front. Public Health 10. https://doi.org/10.3389/fpubh.2022.898043
103. Moreno-Ríos, A.L., Tejeda-Benítez, L.P., Bustillo-Lecompte, C.F., 2022. Sources, characteristics, toxicity, and control of ultrafine particles: An overview. Geoscience Frontiers 13, 101147. https://doi.org/10.1016/j.gsf.2021.101147
104. Münzel, T., Molitor, M., Kuntic, M., Hahad, O., Röösli, M., Engelmann, N., Basner, M., Daiber, A., Sørensen, M., 2024. Transportation Noise Pollution and Cardiovascular Health. Circulation Research 134, 1113–1135. https://doi.org/10.1161/CIRCRESAHA.123.323584
105. Münzel, T., Steven, S., Frenis, K., Lelieveld, J., Hahad, O., Daiber, A., 2020. Environmental Factors Such as Noise and Air Pollution and Vascular Disease. Antioxid Redox Signal 33, 581–601. https://doi.org/10.1089/ars.2020.8090
106. Nassan, F.L., Wang, C., Kelly, R.S., Lasky-Su, J.A., Vokonas, P.S., Koutrakis, P., Schwartz, J.D., 2021. Ambient PM2.5 species and ultrafine particle exposure and their differential metabolomic signatures. Environment International 151, 106447. https://doi.org/10.1016/j.envint.2021.106447
107. Nicholson, S., Baccarelli, A., Prada, D., 2022. Role of brain extracellular vesicles in air pollution-related cognitive impairment and neurodegeneration. Environ Res 204, 112316. https://doi.org/10.1016/j.envres.2021.112316
108. Oberdörster, G., Oberdörster, E., Oberdörster, J., 2005. Nanotoxicology: an emerging discipline evolving from studies of ultrafine particles. Environ Health Perspect 113, 823–839. https://doi.org/10.1289/ehp.7339

109. Olloquequi, J., Díaz-Peña, R., Verdaguer, E., Ettcheto, M., Auladell, C., Camins, A., 2024. From Inhalation to Neurodegeneration: Air Pollution as a Modifiable Risk Factor for Alzheimer's Disease. International Journal of Molecular Sciences 25, 6928. https://doi.org/10.3390/ijms25136928
110. Olstrup, H., Mohamed, H.A.S., Honoré, J., Schullehner, J., Sigsgaard, T., Forsberg, B., Oudin, A., 2024. Air pollution exposure and inflammatory bowel disease: a systematic literature review of epidemiological and mechanistic studies. Front. Environ. Health 3. https://doi.org/10.3389/fenvh.2024.1463016
111. Orr, G.A., Chrisler, W.B., Cassens, K.J., Tan, R., Tarasevich, B.J., Markillie, L.M., Zangar, R.C., Thrall, B.D., 2011. Cellular recognition and trafficking of amorphous silica nanoparticles by macrophage scavenger receptor A. Nanotoxicology 5, 296–311. https://doi.org/10.3109/17435390.2010.513836
112. O'Driscoll, C.A., Gallo, M.E., Hoffmann, E.J., Fechner, J.H., Schauer, J.J., Bradfield, C.A., Mezrich, J.D., 2018. Polycyclic aromatic hydrocarbons (PAHs) present in ambient urban dust drive proinflammatory T cell and dendritic cell responses via the aryl hydrocarbon receptor (AHR) in vitro. PLOS ONE 13, e0209690. https://doi.org/10.1371/journal.pone.0209690
113. Pardo, M., Qiu, X., Zimmermann, R., Rudich, Y., 2020. Particulate Matter Toxicity Is Nrf2 and Mitochondria Dependent: The Roles of Metals and Polycyclic Aromatic Hydrocarbons. Chem Res Toxicol 33, 1110–1120. https://doi.org/10.1021/acs.chemrestox.0c00007
114. Peixoto, M.S., de Oliveira Galvão, M.F., Batistuzzo de Medeiros, S.R., 2017. Cell death pathways of particulate matter toxicity. Chemosphere 188, 32–48. https://doi.org/10.1016/j.chemosphere.2017.08.076
115. Peng, K.-T., Liu, J., Chiang, Y.-C., Chen, P.-C., Chiang, M.-H., Shih, H.-N., Chang, P.-J., Lee, C.-W., 2019. Particulate matter exposure aggravates osteoarthritis severity. Clin Sci (Lond) 133, 2171–2187. https://doi.org/10.1042/CS20190458
116. Peters, A., 2023. Ambient air pollution and Alzheimer's disease: the role of the composition of fine particles. Proceedings of the National Academy of Sciences 120, e2220028120. https://doi.org/10.1073/pnas.2220028120
117. Piyadasa, H., Hemshekhar, M., Carlsten, C., Mookherjee, N., 2018. Inhaled Diesel Exhaust Decreases the Antimicrobial Peptides α-Defensin and S100A7 in Human Bronchial Secretions. Am J Respir Crit Care Med 197, 1358–1361. https://doi.org/10.1164/rccm.201708-1714LE
118. Rahmatinia, M., Mohseni-Bandpei, A., Khodagholi, F., Abdollahifar, M.-A., Amouei Torkmahalleh, M., Hassani Moghaddam, M., Hopke, P.K., Ghavimehr, E., Bazzazpour, S., Shahsavani, A., 2024. Exposure to different PM2.5 extracts induces gliosis and changes behavior in male rats similar to autism spectrum disorders features. Environ Pollut 340, 122804. https://doi.org/10.1016/j.envpol.2023.122804
119. Rajagopalan, S., Brook, R.D., Salerno, P.R.V.O., Bourges-Sevenier, B., Landrigan, P., Nieuwenhuijsen, M.J., Munzel, T., Deo, S.V., Al-Kindi, S., 2024. Air pollution exposure and cardiometabolic risk. The Lancet Diabetes & Endocrinology 12, 196–208. https://doi.org/10.1016/S2213-8587(23)00361-3
120. Rao, X., Patel, P., Puett, R., Rajagopalan, S., 2015. Air Pollution as a Risk Factor for Type 2 Diabetes. Toxicological Sciences 143, 231–241. https://doi.org/10.1093/toxsci/kfu250
121. Rao, X., Zhong, J., Brook, R.D., Rajagopalan, S., 2018. Effect of Particulate Matter Air Pollution on Cardiovascular Oxidative Stress Pathways. Antioxidants & Redox Signaling 28, 797–818. https://doi.org/10.1089/ars.2017.7394
122. Rao, X., Zhong, J., Maiseyeu, A., Gopalakrishnan, B., Villamena, F.A., Chen, L.-C., Harkema, J.R., Sun, Q., Rajagopalan, S., 2014. CD36-dependent 7-ketocholesterol accumulation in macrophages mediates progression of atherosclerosis in response to chronic air pollution exposure. Circ Res 115, 770–780. https://doi.org/10.1161/CIRCRESAHA.115.304666

123. Reisetter, A.C., Stebounova, L.V., Baltrusaitis, J., Powers, L., Gupta, A., Grassian, V.H., Monick, M.M., 2011. Induction of Inflammasome-dependent Pyroptosis by Carbon Black Nanoparticles *. Journal of Biological Chemistry 286, 21844–21852. https://doi.org/10.1074/jbc.M111.238519
124. Reynolds, P.R., Wasley, K.M., Allison, C.H., 2011. Diesel particulate matter induces receptor for advanced glycation end-products (RAGE) expression in pulmonary epithelial cells, and RAGE signaling influences NF-κB-mediated inflammation. Environ Health Perspect 119, 332–336. https://doi.org/10.1289/ehp.1002520
125. Ribeiro, H., Guimarães, F., Duque, L., Noronha, F., Abreu, I., 2015. Characterisation of particulate matter on airborne pollen grains. Environ Pollut 206, 7–16. https://doi.org/10.1016/j.envpol.2015.06.015
126. Robertson, S., Thomson, A.L., Carter, R., Stott, H.R., Shaw, C.A., Hadoke, P.W.F., Newby, D.E., Miller, M.R., Gray, G.A., 2014. Pulmonary diesel particulate increases susceptibility to myocardial ischemia/reperfusion injury via activation of sensory TRPV1 and β1 adrenoreceptors. Part Fibre Toxicol 11, 12. https://doi.org/10.1186/1743-8977-11-12
127. Rubio, C., López-Landa, A., Serrano-García, N., Romo-Parra, H., Rubio-Osornio, M., 2025. Impact of Particulate Matter 2.5 on Neurological Diseases: Insights Into Pathophysiological and Molecular Mechanisms. J Toxicol 2025, 5752904. https://doi.org/10.1155/jt/5752904
128. Rui, W., Guan, L., Zhang, F., Zhang, W., Ding, W., 2016. PM2.5-induced oxidative stress increases adhesion molecules expression in human endothelial cells through the ERK/AKT/NF-κB-dependent pathway. Journal of Applied Toxicology 36, 48–59. https://doi.org/10.1002/jat.3143
129. Rumelhard, M., Ramgolam, K., Hamel, R., Marano, F., Baeza-Squiban, A., 2007. Expression and role of EGFR ligands induced in airway cells by PM2.5 and its components. European Respiratory Journal 30, 1064–1073. https://doi.org/10.1183/09031936.00085907
130. Sagheer, U., Al-Kindi, S., Abohashem, S., Phillips, C.T., Rana, J.S., Bhatnagar, A., Gulati, M., Rajagopalan, S., Kalra, D.K., 2024. Environmental Pollution and Cardiovascular Disease: Part 1 of 2: Air Pollution. JACC Adv 3, 100805. https://doi.org/10.1016/j.jacadv.2023.100805
131. Sahu, B., Mackos, A.R., Floden, A.M., Wold, L.E., Combs, C.K., 2021. Particulate Matter Exposure Exacerbates Amyloid-β Plaque Deposition and Gliosis in APP/PS1 Mice. J Alzheimers Dis 80, 761–774. https://doi.org/10.3233/JAD-200919
132. Scheers, H., Jacobs, L., Casas, L., Nemery, B., Nawrot, T.S., 2015. Long-Term Exposure to Particulate Matter Air Pollution Is a Risk Factor for Stroke. Stroke 46, 3058–3066. https://doi.org/10.1161/STROKEAHA.115.009913
133. Schlesinger, R.B., 2007. The health impact of common inorganic components of fine particulate matter (PM2.5) in ambient air: a critical review. Inhal Toxicol 19, 811–832. https://doi.org/10.1080/08958370701402382
134. Schraufnagel, D.E., Balmes, J.R., Cowl, C.T., Matteis, S.D., Jung, S.-H., Mortimer, K., Perez-Padilla, R., Rice, M.B., Riojas-Rodriguez, H., Sood, A., Thurston, G.D., To, T., Vanker, A., Wuebbles, D.J., 2019. Air Pollution and Noncommunicable Diseases: A Review by the Forum of International Respiratory Societies' Environmental Committee, Part 2: Air Pollution and Organ Systems. CHEST 155, 417–426. https://doi.org/10.1016/j.chest.2018.10.041
135. Seneviratne, A.N., Miller, M.R., 2025. Air pollution and atherosclerosis. Atherosclerosis 406, 119240. https://doi.org/10.1016/j.atherosclerosis.2025.119240
136. Shaw, C.A., Robertson, S., Miller, M.R., Duffin, R., Tabor, C.M., Donaldson, K., Newby, D.E., Hadoke, P.W.F., 2011. Diesel exhaust particulate--exposed macrophages cause marked endothelial cell activation. Am J Respir Cell Mol Biol 44, 840–851. https://doi.org/10.1165/rcmb.2010-0011OC
137. Shkirkova, K., Demetriou, A.N., Sizdahkhani, S., Lamorie-Foote, K., Zhang, H., Morales, M., Chen, S., Zhao, L., Diaz, A., Godoy-Lugo, J.A., Zhou, B., Zhang, N., Li, A., Mack, Wendy J., Sioutas, C., Thorwald, M.A., Finch, C.E., Pike, C., Mack, William J., 2024. Microglial TLR4

Mediates White Matter Injury in a Combined Model of Diesel Exhaust Exposure and Cerebral Hypoperfusion. Stroke 55, 1090–1093. https://doi.org/10.1161/STROKEAHA.124.046412
138. Shoenfelt, J., Mitkus, R.J., Zeisler, R., Spatz, R.O., Powell, J., Fenton, M.J., Squibb, K.A., Medvedev, A.E., 2009. Involvement of TLR2 and TLR4 in inflammatory immune responses induced by fine and coarse ambient air particulate matter. J Leukoc Biol 86, 303–312. https://doi.org/10.1189/jlb.1008587
139. Sioutas, C., Delfino, R.J., Singh, M., 2005. Exposure assessment for atmospheric ultrafine particles (UFPs) and implications in epidemiologic research. Environ Health Perspect 113, 947–955. https://doi.org/10.1289/ehp.7939
140. Steerenberg, P.A., van Amelsvoort, L., Lovik, M., Hetland, R.B., Alberg, T., Halatek, T., Bloemen, H.J.T., Rydzynski, K., Swaen, G., Schwarze, P., Dybing, E., Cassee, F.R., 2006. Relation between sources of particulate air pollution and biological effect parameters in samples from four European cities: an exploratory study. Inhal Toxicol 18, 333–346. https://doi.org/10.1080/08958370500515913
141. Stone, V., Miller, M.R., Clift, M.J.D., Elder, A., Mills, N.L., Møller, P., Schins, R.P.F., Vogel, U., Kreyling, W.G., Alstrup Jensen, K., Kuhlbusch, T.A.J., Schwarze, P.E., Hoet, P., Pietroiusti, A., De Vizcaya-Ruiz, A., Baeza-Squiban, A., Teixeira, J.P., Tran, C.L., Cassee, F.R., 2017. Nanomaterials Versus Ambient Ultrafine Particles: An Opportunity to Exchange Toxicology Knowledge. Environmental Health Perspectives 125, 106002. https://doi.org/10.1289/EHP424
142. Sun, Q., Wang, A., Jin, X., Natanzon, A., Duquaine, D., Brook, R.D., Aguinaldo, J.-G.S., Fayad, Z.A., Fuster, V., Lippmann, M., Chen, L.C., Rajagopalan, S., 2005. Long-term air pollution exposure and acceleration of atherosclerosis and vascular inflammation in an animal model. JAMA 294, 3003–3010. https://doi.org/10.1001/jama.294.23.3003
143. Sun, Q., Yue, P., Kirk, R.I., Wang, A., Moatti, D., Jin, X., Lu, B., Schecter, A.D., Lippmann, M., Gordon, T., Chen, L.C., Rajagopalan, S., 2008a. Ambient air particulate matter exposure and tissue factor expression in atherosclerosis. Inhal Toxicol 20, 127–137. https://doi.org/10.1080/08958370701821482
144. Sun, Q., Yue, P., Ying, Z., Cardounel, A.J., Brook, R.D., Devlin, R., Hwang, J.-S., Zweier, J.L., Chen, L.C., Rajagopalan, S., 2008b. Air pollution exposure potentiates hypertension through reactive oxygen species-mediated activation of Rho/ROCK. Arterioscler Thromb Vasc Biol 28, 1760–1766. https://doi.org/10.1161/ATVBAHA.108.166967
145. Sydlik, U., Bierhals, K., Soufi, M., Abel, J., Schins, R.P.F., Unfried, K., 2006. Ultrafine carbon particles induce apoptosis and proliferation in rat lung epithelial cells via specific signaling pathways both using EGF-R. American Journal of Physiology-Lung Cellular and Molecular Physiology 291, L725–L733. https://doi.org/10.1152/ajplung.00131.2006
146. Tabor, C.M., Shaw, C.A., Robertson, S., Miller, M.R., Duffin, R., Donaldson, K., Newby, D.E., Hadoke, P.W.F., 2016. Platelet activation independent of pulmonary inflammation contributes to diesel exhaust particulate-induced promotion of arterial thrombosis. Part Fibre Toxicol 13, 6. https://doi.org/10.1186/s12989-016-0116-x
147. Thangavel, P., Park, D., Lee, Y.-C., 2022. Recent Insights into Particulate Matter (PM2.5)-Mediated Toxicity in Humans: An Overview. Int J Environ Res Public Health 19, 7511. https://doi.org/10.3390/ijerph19127511
148. Traboulsi, H., Guerrina, N., Iu, M., Maysinger, D., Ariya, P., Baglole, C.J., 2017. Inhaled Pollutants: The Molecular Scene behind Respiratory and Systemic Diseases Associated with Ultrafine Particulate Matter. International Journal of Molecular Sciences 18, 243. https://doi.org/10.3390/ijms18020243
149. Unosson, J., Kabéle, M., Boman, C., Nyström, R., Sadiktsis, I., Westerholm, R., Mudway, I.S., Purdie, E., Raftis, J., Miller, M.R., Mills, N.L., Newby, D.E., Blomberg, A., Sandström, T., Bosson, J.A., 2021. Acute cardiovascular effects of controlled exposure to dilute Petrodiesel and

biodiesel exhaust in healthy volunteers: a crossover study. Part Fibre Toxicol 18, 22. https://doi.org/10.1186/s12989-021-00412-3
150. Valavanidis, A., Vlachogianni, T., Fiotakis, K., Loridas, S., 2013. Pulmonary oxidative stress, inflammation and cancer: respirable particulate matter, fibrous dusts and ozone as major causes of lung carcinogenesis through reactive oxygen species mechanisms. Int J Environ Res Public Health 10, 3886–3907. https://doi.org/10.3390/ijerph10093886
151. Vilas-Boas, V., Chatterjee, N., Carvalho, A., Alfaro-Moreno, E., 2024. Particulate matter-induced oxidative stress – Mechanistic insights and antioxidant approaches reported in in vitro studies. Environmental Toxicology and Pharmacology 110, 104529. https://doi.org/10.1016/j.etap.2024.104529
152. Volk, H.E., Lurmann, F., Penfold, B., Hertz-Picciotto, I., McConnell, R., 2013. Traffic-related air pollution, particulate matter, and autism. JAMA Psychiatry 70, 71–77. https://doi.org/10.1001/jamapsychiatry.2013.266
153. Wang, M., Niu, Yong, Liu, Q., Yang, P., Wu, M., Wu, R., Shi, B., Chen, J., Wang, J., Du, Z., Pang, Y., Bao, L., Niu, Yujie, Zhang, R., 2025. Carbon black induced pulmonary fibrosis through piR-713551/PIWIL4 targeting THBS2 signal pathway. J Environ Sci (China) 155, 409–422. https://doi.org/10.1016/j.jes.2024.05.017
154. Wang, T., Lang, G.D., Moreno-Vinasco, L., Huang, Y., Goonewardena, S.N., Peng, Y.-J., Svensson, E.C., Natarajan, V., Lang, R.M., Linares, J.D., Breysse, P.N., Geyh, A.S., Samet, J.M., Lussier, Y.A., Dudley, S., Prabhakar, N.R., Garcia, J.G.N., 2012. Particulate matter induces cardiac arrhythmias via dysregulation of carotid body sensitivity and cardiac sodium channels. Am J Respir Cell Mol Biol 46, 524–531. https://doi.org/10.1165/rcmb.2011-0213OC
155. Wang, Y., Li, C., Zhang, X., Kang, X., Li, Y., Zhang, W., Chen, Y., Liu, Y., Wang, W., Ge, M., Du, L., 2021. Exposure to PM2.5 aggravates Parkinson's disease via inhibition of autophagy and mitophagy pathway. Toxicology 456, 152770. https://doi.org/10.1016/j.tox.2021.152770
156. Weldy, C.S., Liu, Y., Liggitt, H.D., Chin, M.T., 2014. In utero exposure to diesel exhaust air pollution promotes adverse intrauterine conditions, resulting in weight gain, altered blood pressure, and increased susceptibility to heart failure in adult mice. PLoS One 9, e88582. https://doi.org/10.1371/journal.pone.0088582
157. Wen, J., Zhang, J., Zhang, H., Zhang, N., Lei, R., Deng, Y., Cheng, Q., Li, H., Luo, P., 2024. Large-scale genome-wide association studies reveal the genetic causal etiology between air pollutants and autoimmune diseases. Journal of Translational Medicine 22, 392. https://doi.org/10.1186/s12967-024-04928-y
158. Wilker, E.H., Osman, M., Weisskopf, M.G., 2023. Ambient air pollution and clinical dementia: systematic review and meta-analysis. BMJ 381, e071620. https://doi.org/10.1136/bmj-2022-071620
159. Wold, L.E., Ying, Z., Hutchinson, K.R., Velten, M., Gorr, M.W., Velten, C., Youtz, D.J., Wang, A., Lucchesi, P.A., Sun, Q., Rajagopalan, S., 2012. Cardiovascular remodeling in response to long-term exposure to fine particulate matter air pollution. Circ Heart Fail 5, 452–461. https://doi.org/10.1161/CIRCHEARTFAILURE.112.966580
160. Xia, M., Viera-Hutchins, L., Garcia-Lloret, M., Rivas, M.N., Wise, P., McGhee, S.A., Chatila, Z.K., Daher, N., Sioutas, C., Chatila, T.A., 2015. Vehicular exhaust particles promote allergic airway inflammation through an aryl hydrocarbon receptor–notch signaling cascade. Journal of Allergy and Clinical Immunology 136, 441–453. https://doi.org/10.1016/j.jaci.2015.02.014
161. Xia, T., Zhu, Y., Mu, L., Zhang, Z.-F., Liu, S., 2016. Pulmonary diseases induced by ambient ultrafine and engineered nanoparticles in twenty-first century. Natl Sci Rev 3, 416–429. https://doi.org/10.1093/nsr/nww064
162. Xiong, Q., Tian, X., Xu, C., Ma, B., Li, W., Xia, Y., Liu, W., Sun, B., Ru, Q., Shu, X., 2024. Mediation of PM2.5-induced cytotoxicity: the role of P2X7 receptor in NR8383 cells. Int J Environ Health Res 34, 1602–1614. https://doi.org/10.1080/09603123.2023.2230920

163. Xu, H., Wang, T., Liu, S., Brook, R.D., Feng, B., Zhao, Q., Song, X., Yi, T., Chen, J., Zhang, Y., Wang, Y., Zheng, L., Rajagopalan, S., Li, J., Huang, W., 2019. Extreme Levels of Air Pollution Associated With Changes in Biomarkers of Atherosclerotic Plaque Vulnerability and Thrombogenicity in Healthy Adults. Circ Res 124, e30–e43. https://doi.org/10.1161/CIRCRESAHA.118.313948
164. Xu, X., Kherada, N., Hong, X., Quan, C., Zheng, L., Wang, A., Wold, L.E., Lippmann, M., Chen, L.C., Rajagopalan, S., Sun, Q., 2009. Diesel exhaust exposure induces angiogenesis. Toxicol Lett 191, 57–68. https://doi.org/10.1016/j.toxlet.2009.08.006
165. Xu, X., Yavar, Z., Verdin, M., Ying, Z., Mihai, G., Kampfrath, T., Wang, A., Zhong, M., Lippmann, M., Chen, L.-C., Rajagopalan, S., Sun, Q., 2010. Effect of early particulate air pollution exposure on obesity in mice: role of p47phox. Arterioscler Thromb Vasc Biol 30, 2518–2527. https://doi.org/10.1161/ATVBAHA.110.215350
166. Xu, X., Zhang, J., Yang, X., Zhang, Y., Chen, Z., 2020. The Role and Potential Pathogenic Mechanism of Particulate Matter in Childhood Asthma: A Review and Perspective. J Immunol Res 2020, 8254909. https://doi.org/10.1155/2020/8254909
167. Yang, Jinho, Kim, E.K., Park, H.J., McDowell, A., Kim, Y.-K., 2020. The impact of bacteria-derived ultrafine dust particles on pulmonary diseases. Exp Mol Med 52, 338–347. https://doi.org/10.1038/s12276-019-0367-3
168. Yang, Junling, Wise, L., Fukuchi, K., 2020. TLR4 Cross-Talk With NLRP3 Inflammasome and Complement Signaling Pathways in Alzheimer's Disease. Front. Immunol. 11. https://doi.org/10.3389/fimmu.2020.00724
169. Ying, Z., Yue, P., Xu, X., Zhong, M., Sun, Q., Mikolaj, M., Wang, A., Brook, R.D., Chen, L.C., Rajagopalan, S., 2009. Air pollution and cardiac remodeling: a role for RhoA/Rho-kinase. Am J Physiol Heart Circ Physiol 296, H1540-1550. https://doi.org/10.1152/ajpheart.01270.2008
170. Yu, W., Xu, R., Ye, T., Abramson, M.J., Morawska, L., Jalaludin, B., Johnston, F.H., Henderson, S.B., Knibbs, L.D., Morgan, G.G., Lavigne, E., Heyworth, J., Hales, S., Marks, G.B., Woodward, A., Bell, M.L., Samet, J.M., Song, J., Li, S., Guo, Y., 2024. Estimates of global mortality burden associated with short-term exposure to fine particulate matter (PM2·5). Lancet Planet Health 8, e146–e155. https://doi.org/10.1016/S2542-5196(24)00003-2
171. Yuan, X., Yang, Y., Liu, C., Tian, Y., Xia, D., Liu, Z., Pan, L., Xiong, M., Xiong, J., Meng, L., Zhang, Zhaohui, Ye, K., Jiang, H., Zhang, Zhentao, 2022. Fine Particulate Matter Triggers α-Synuclein Fibrillization and Parkinson-like Neurodegeneration. Mov Disord 37, 1817–1830. https://doi.org/10.1002/mds.29181
172. Zhang, J., Chen, Z., Shan, D., Wu, Y., Zhao, Y., Li, C., Shu, Y., Linghu, X., Wang, B., 2024. Adverse effects of exposure to fine particles and ultrafine particles in the environment on different organs of organisms. Journal of Environmental Sciences 135, 449–473. https://doi.org/10.1016/j.jes.2022.08.013
173. Zhang, R.-D., Chen, C., Wang, P., Fang, Y., Jiang, L.-Q., Fang, X., Zhao, Y., Ni, J., Wang, D.-G., Pan, H.-F., 2023. Air pollution exposure and auto-inflammatory and autoimmune diseases of the musculoskeletal system: a review of epidemiologic and mechanistic evidence. Environ Geochem Health 45, 4087–4105. https://doi.org/10.1007/s10653-023-01495-x
174. Zhang, Xiaodi, Liu, H., Wu, X., Jia, L., Gadhave, K., Wang, L., Zhang, K., Li, H., Chen, R., Kumbhar, R., Wang, N., Terrillion, C.E., Kang, B.G., Bai, B., Park, M., Denna, M.C.F., Zhang, S., Zheng, W., Ye, D., Rong, X., Yang, L., Niu, L., Ko, H.S., Peng, W., Jin, L., Ying, M., Rosenthal, L.S., Nauen, D.W., Pantelyat, A., Kaur, M., Irene, K., Shi, L., Feleke, R., García-Ruiz, S., Ryten, M., Dawson, V.L., Dominici, F., Weber, R.J., Zhang, Xuan, Liu, P., Dawson, T.M., Han, S., Mao, X., 2025. Lewy body dementia promotion by air pollutants. Science 389, eadu4132. https://doi.org/10.1126/science.adu4132
175. Zhong, J., Zhao, G., Edwards, S., Tran, J., Rajagopalan, S., Rao, X., 2023. Particulate air pollution exaggerates diet-induced insulin resistance through NLRP3 inflammasome in mice. Environ Pollut 328, 121603. https://doi.org/10.1016/j.envpol.2023.121603

176. Øvrevik, J., Refsnes, M., Låg, M., Holme, J.A., Schwarze, P.E., 2015. Activation of Proinflammatory Responses in Cells of the Airway Mucosa by Particulate Matter: Oxidant- and Non-Oxidant-Mediated Triggering Mechanisms. Biomolecules 5, 1399–1440. https://doi.org/10.3390/biom5031399
