# Supplementary material for: ExposomeMap-PM: fine and ultrafine particle-induced toxicity - from adverse outcome pathways to systems biomedicine disease models
Source: Front Toxicol. 2026 Oct 27;8:1951777. doi: 10.3389/ftox.2026.1951777 (PMC13749717; doi:10.3389/ftox.2026.1951777)
Supplement: Supplementary file 3 [file DataSheet3.pdf]

Supplementary Table S5.1. Overall mapping and enrichment of the proof-of-concept omics overlays on ExposomeMap-PM nodes. Differential expression is defined as nominal $p < 0.05$ in the complete feature table of each dataset; unique gene symbols were counted once and map membership was assessed against the 144 HGNC-annotated nodes in Supplementary File S4. Enrichment is the ratio of the observed number of differentially expressed map nodes to the mean of 10,000 size-matched random gene sets drawn from the same tested background; the p-value is the corresponding empirical permutation p-value.

Metric	T1 transcriptomics	P1 proteomics
Unique gene symbols tested (complete table)	18,606	5,739
ExposomeMap-PM nodes represented	131	79
Differentially expressed features ($p < 0.05$)	3,312	1,160
Differentially expressed map nodes	35	18
Differentially expressed fraction of represented nodes	35/131 (26.7%)	18/79 (22.8%)
Matched random-set mean (SD)	23.30 (4.38)	15.97 (3.54)
Enrichment	1.50-fold	1.13-fold
Empirical permutation p-value	0.0074	0.3199

Supplementary Table S5.2. Block-level overlay statistics for the proof-of-concept omics datasets. Values are the number of nominally differentially expressed map nodes ($p < 0.05$) out of the map nodes of that block represented in the dataset, with the corresponding percentage, the mean absolute log fold-change of the block's map nodes, and the one-sided hypergeometric p-value for the block's differentially expressed count against the complete tested background of that dataset. All five blocks contain differentially expressed nodes in both datasets. The per-block tests are reported for completeness and are inherently underpowered at these block sizes (3 to 56 represented nodes) and for a single reference particle type and a 3-day exposure; they are not intended as independent evidence for or against individual blocks.

Block	T1: DE / represented nodes	T1: mean |logFC|	T1: p	P1: DE / represented nodes	P1: mean |logFC|	P1: p
Receptor activation	8/32 (25.0%)	0.214	0.197	3/17 (17.6%)	0.375	0.698
Phagocytic recognition	11/56 (19.6%)	0.274	0.412	8/33 (24.2%)	0.259	0.346
Ion channel perturbation	5/13 (38.5%)	0.237	0.065	1/3 (33.3%)	0.267	0.492
Surface catalytic interactions	4/13 (30.8%)	0.319	0.188	1/5 (20.0%)	0.226	0.677
Antioxidant and detoxification	8/26 (30.8%)	0.195	0.077	6/26 (23.1%)	0.157	0.433
