# Supplementary material for: ExposomeMap-PM: fine and ultrafine particle-induced toxicity - from adverse outcome pathways to systems biomedicine disease models
Source: Front Toxicol. 2026 Oct 27;8:1951777. doi: 10.3389/ftox.2026.1951777 (PMC13749717; doi:10.3389/ftox.2026.1951777)
Supplement: Supplementary file 4 [file DataSheet4.pdf]

Supplementary Table S6. Examples of diseases relevant to PM2.5 and UFP exposure. Disease Ontology database (https://disease-ontology.org) was used for identifying diseases, for example for Parkinson's disease it is DOID:14330 (https://disease-ontology.org/?id=DOID:14330) and from there can be linked to other disease databases such as ICD10CM, ICD9CM, MESH and OMIM. Evidence type: h, human; m, mouse; r, rat; epi, epidemiological; exp, in vivo experimental; in vitro, in vitro experimental; review, review article; meta, systematic review/meta-analysis.

Disease/ Outcome	Disease ID	Key mechanisms	References	Evidence type
**Cardiovascular**				
Ischemic heart disease	DOID:5844	Coronary oxidative stress and endothelial dysfunction, cardiomyocyte injury, and pulmonary sensory receptor–driven autonomic/adrenergic imbalance contribute to ischemic cardiac dysfunction.	Brook 2008 PMID:18691154 Robertson 2014 PMID:24568236 Miller 2020 PMID:31923583	r-exp; review
Cerebrovascular diseases	DOID:6713	Oxidative stress and vascular inflammation drive cerebrovascular dysfunction and ischemic injury via NLRP3 inflammasome activation; endothelial dysfunction and IL-6-mediated fibrinogen upregulation sustain a prothrombotic state leading to cerebral ischemia.	Scheers 2015 PMID:26463695 Li PMID: 3615076 Rubio 2025 PMID:41498103 Chen 2022 10.1016/j.envpol.2022.119827	meta; review
Hypertension	DOID:10763	Vascular oxidative stress and autonomic imbalance elevate arterial pressure and stiffness; ROS-mediated RhoA/ROCK activation potentiates vasoconstriction; PM-induced DNA hypomethylation activates inflammatory and vascular pathways	Mills 2008 PMID:19029991 Li 2017 PMID:28808144 Ying 2009 PMID:19286943	h-exp; m-exp; review
Heart Failure	DOID:6000	Endothelial dysfunction, autonomic imbalance, and pro-thrombotic shifts activate redox-sensitive pathways (NF-κB, Nrf2) and inflammasome signalling (NLRP3), driving mitochondrial dysfunction, impaired myocardial contractility, and arrhythmogenic remodelling	Brook 2008 PMID:18691154 Weldy 2014 PMID:24533117 Münzel 2020 PMID:32245334 An 2019 PMID:31354731	m-exp; review
Heart conduction disease	DOID:10273	Autonomic dysregulation and oxidative stress disrupt cardiac conduction via carotid body ion channel dysregulation (Na^+/K^+ channels), SCN5A nitration, and TRPA1-mediated sympathetic activation	Mandaglio-Collados 2023 PMID:37023806 Fiordelisi PMID: 28303426 Wang 2012 PMID:22108299 Hazari 2011 PMID:21377951	m-exp; r-exp; review
Atherosclerosis	DOID:1936	Endothelial activation, macrophage lipid uptake and foam cell formation drive	Bevan 2021 PMID:34417890	m-exp; review

		plaque instability; ROS-mediated aortic oxidative stress and impaired HDL protective capacity accelerate atherosclerotic progression	Seneviratne 2025 PMID:40411956 Sun 2005 PMID:16414948 Araujo 2008 PMID:18202315	
Chronic kidney disease	DOID:784	Endothelial dysfunction, ROS-induced podocyte injury, and microvascular inflammation; GPX4-regulated ferroptosis and epithelial-to-mesenchymal transition in tubular cells	Hsu PMID:30878646 Bowe 2018 PMID:28935655 Lin 2025 PMID:39631540	h-epi; r-exp; h-in vitro
**Lung disease**				
Pneumonia	DOID:552	PM exposure downregulates antimicrobial peptides (α-defensin, S100A7, hBD-2) via ROS-mediated epithelial senescence, impairing airway antibacterial defence and promoting bacterial invasion	Piyadasa 2018 PMID:29244524 Chen 2018 PMID:29310642	h-exp; h-in vitro
Pulmonary fibrosis	DOID:3770	Macrophage lactate production stabilises TGF-β1 via CHIP suppression, driving epithelial-to-mesenchymal transition and lung fibrosis	Wang 2025 PMID:40246476 Liu 2025 PMID:40759348	m-exp; m-in vitro; h-in vitro
Asthma	DOID:2841	Epithelial injury and Th2-mediated inflammation amplify bronchial hyperreactivity and remodelling; EGFR-PI3Kα-AKT/ERK signalling drives mucus hypersecretion; DEP-induced CXC chemokines recruit neutrophils and augment airway inflammation	Xu 2020 PMID:32411804 Fortoul 2011 DOI:10.5772/17766 Kim 2011 PMID:21967814	m-exp; review
COPD	DOID:3083	Chronic oxidative stress and inflammation cause airway remodelling and emphysema; NF-κB/Nrf2 pathways and downstream cytokine, mucus, and remodeling responses	Kaur 2022 PMID:35710643 Fortoul 2011 DOI:10.5772/17766 Cachon 2014 PMID:24333687 https://doi.org/10.1038/s12276-020-0394-0	h-in vitro; review
Lung Cancer	DOID:1324	DNA damage, chronic inflammation, and epigenetic dysregulation; DEP-induced immunosuppressive tumor microenvironment promoting KRAS-driven adenocarcinoma progression	Fortoul 2011 DOI:10.5772/17766 Valavanidis 2013 PMID: 23985773 Hill 2023 (human, mice) PMID:37020004 Delhez 2025 PMID:41274118	h-epi; m-exp; review
**Neurological disorders**				
Alzheimer's disease	DOID:10652	Chronic exposure induces oxidative stress, microglial activation, gliosis, amyloid	Peters 2023 PMID:36626559	m-exp; review

		pathology, tau aggregation, mitochondrial dysfunction, and excitotoxicity	Rubio 2025 PMID:41498103 Olloquequi 2024 PMID:39000036 Liu 2024 PMID:39133647 Sahu 2021 PMID:33554902	
Parkinson's disease	DOID:14330	Neuroinflammation, oxidative stress, and α-synuclein fibrillization drive dopaminergic neuronal loss; CpG hypermethylation silences PINK1/DJ-1; suppressed mitophagy promotes mitochondria-mediated apoptosis	Calderón-Garciduеñas 2020 PMID:32955466 Rubio 2025 PMID:41498103 Wang 2021 PMID:33823232 Du 2025 PMID:40155616 Yuan 2022 PMID:36054165	m-exp; r-exp; h-in vitro; r-in vitro; review
Cognitive Impairment & dementia	DOID:1561	Chronic neuroinflammation, oxidative stress, microvascular compromise, and hippocampal neurogenesis inhibition; BACE1 upregulation promotes amyloidogenic APP processing	Wilker 2023 PMID:37019461 Rubio 2025 PMID:41498103	meta; review
Lewy body dementia	DOID: 12217	Alpha-synuclein aggregation and propagation, neurodegeneration, brain atrophy, and cognitive decline	Xiaodi Zhang 2025 PMID: 40906862	h-epi; m-exp; m-in vitro
Attention deficit hyperactivity disorder	DOID:1094	Microglial hyperactivation, dopamine-pathway disruption, impaired myelination and oxidative stress affecting fronto-striatal circuit development	Ahmad 2024 PMID:39544605	meta
Autism spectrum disorder	DOID:0060041	Neuroinflammation, glial activation, synaptic pruning disruption, mitochondrial dysfunction, and epigenetic shifts impair cortical circuits; oxytocin reduction across limbic, prefrontal, and cerebellar regions contributes to social behaviour deficits	Dutheil 2021 PMID:33714060 Rahmatinia 2024 PMID:37907193	r-exp; meta
**Infectious diseases**				
Influenza	DOID:8469	UFP and oxidized lipids degrade MARCO, suppressing IFN-α/β antiviral responses; PM2.5 further impairs innate immunity via NLRP3 inflammasome inhibition and IFN-β suppression, amplifying NF-κB-driven inflammation and viral replication	Ghosh 2011 PMID:21562316 Liu 2023 PMID:37935887 Tao 2020 PMID:32717666	m-exp; m-in vitro; h-in vitro
Bacterial pneumonia	DOID:874	MARCO downregulation weakens MyD88–NF-κB antibacterial responses; CAP exposure increases S. pneumoniae binding while	Arredouani 2004 PMID:15263032 Zhou 2007 PMID:17079778	m-exp; m-in vitro

		impairing internalization, compromising bacterial clearance		
**Skin diseases**				
Atopic dermatitis, eczema, dermatitis	DOID:2723	Metal-driven ROS generation causes lipid oxidation and NF-κB/COX-2 activation; AhR–NF-κB signalling disrupts epidermal barrier and enhances Th2 inflammation; CAPs penetrate skin tissue and trigger apoptosis	Kim 2022 PMID:35697899 Magnani 2016 PMID26507108	h-in vitro
Psoriasis	DOID:8893	ROS production and AhR/NF-κB/p38 MAPK signalling in keratinocytes, upregulating S100A7/S100A8 and MMP1 while downregulating barrier proteins (keratin-10, DSC1, CLDN1), driving psoriatic gene expression and epidermal dysfunction	Kim 2017 PMID:28341207 Magnani 2016 PMID26507108	h-in vitro
**Metabolic diseases**				
Type 2 diabetes mellitus	DOID:9352	Systemic inflammation, oxidative stress, and insulin signalling impairment; adipokine dysregulation and mitochondrial dysfunction in adipose tissue impairing brown fat thermogenesis and glucose homeostasis	He 2017 PMID:28122165 Guo 2024 PMID:38489900 Xu 2011 PMID:21873646	h-epi; m-exp; meta
Non-alcoholic fatty liver disease	DOID:0080208	Oxidative stress and AhR/Nrf2 imbalance drive lipid accumulation; TLR4/NF-κB/NLRP3 inflammasome activation promotes hepatic inflammation and fibrosis; mitochondrial structural damage amplifies ROS production	Jacopo Iaccarino 2025 PMID:41154457 Fu 2025 PMID:39979029 Liu 2024 PMID:38789588	m-exp; r-exp; review
**Autoimmune disorders**				
Autoimmune diseases	DOID:417	Oxidative stress drives monocyte and dendritic cell activation (CD40, CD80, CD83 upregulation) and T cell effector skewing, promoting autoantibody production and tissue damage	Wen 2024 PMID:38685026 Acosta-Ampudia 2025 PMID:41492411	h-epi; h-in vitro
Rheumatoid Arthritis	DOID:7148	Oxidative stress, AhR/NF-κB activation, and Th17 polarization aggravate joint synovitis in rheumatoid arthritis	Zhang 2023 PMID:36735155	review
Multiple sclerosis	DOID:2377	PM2.5 upregulates CCR6 on CD4+ T cells, promoting IL-1β and IL-6 secretion by innate immune cells and driving chronic neuroinflammation	Mohammadi 2022 PMID:35875044	review
Uveitis	DOID:13141	Th17 cell expansion and IL-17A upregulation drive uveal	Liu 2024 PMID:39232294	m-exp; m-in vitro

		inflammation; PM2.5 induces ferroptosis in CD4+ T cells via GPX4/GSH suppression, Fe^{2+} and lipid ROS accumulation, and HMOX1/TFRC upregulation		
**Degenerative joint diseases**				
Osteoarthritis	DOID:8398	ROS and NOS-driven oxidative stress and DAMP-mediated innate immune activation; PM exacerbates cartilage and bone matrix degradation via dysregulation of osteocalcin, COMP, and collagen turnover	Peng 2019 PMID:31696218; Chan 2025 PMID:40350055	h-epi; r-exp
**Gastrointestinal disorders**				
Inflammatory bowel disease	DOID:0050589	Increased gut permeability and decreased colonic motility; PM2.5-induced microbiota dysbiosis with Bacteroidetes depletion and expansion of pro-inflammatory genera disrupts intestinal homeostasis	Olstrup 2024 DOI:10.3389/fenvh.2024.1463016 Liu 2021 https://doi.org/10.1016/j.ecoenv.2021.111903	m-exp; meta
**Reproductive**				
Male infertility	DOID:12063	Oxidative stress and mitochondrial dysfunction impair sperm quality and hormone balance; DEP disrupts meiotic DSB repair and GnRH signalling, impairing spermatogenesis	Ma 2024 PMID:38135136 Yang 2020 PMID:32574863	h-epi; m-exp
Placental dysfunction / Fetal growth restriction	DOID:5223	Oxidative stress and endocrine disruption impair placental development; in utero DE exposure promotes vascular oxidative stress (3-NT modification) and immune infiltration in placental tissue, reducing placental weight and impairing fetal perfusion	Hooven 2012 PMID:22922820 Weldy 2014 PMID:24533117	h-epi; m-exp
