# Supplementary material for: ExposomeMap-PM: fine and ultrafine particle-induced toxicity - from adverse outcome pathways to systems biomedicine disease models
Source: Front Toxicol. 2026 Oct 27;8:1951777. doi: 10.3389/ftox.2026.1951777 (PMC13749717; doi:10.3389/ftox.2026.1951777)
Supplement: Supplementary file 5 [file DataSheet5.pdf]

Supplementary Table S7. ExposomeMap-PM statistics.

Item	Count
Species	304
Reactions/processes	497
Molecular nodes	236
- Proteins	144
- Genes	25
- mRNAs	24
- Simple chemicals	43
Complexes	10
Phenotypes	35
Particle sources/types	23
MIRIAM fraction - annotated molecular nodes	214 of 236 (91%)
Curated references - Supplementary Files S1, S8	176
Block 1 - Receptor activation	41
Block 2 - Phagocytic recognition	105
Block 3 - Ion channel perturbation	59
Block 4 - Surface catalytic interactions	21
Block 5 - Antioxidant and detoxification	61
