# Supplementary material for: ExposomeMap-PM: fine and ultrafine particle-induced toxicity - from adverse outcome pathways to systems biomedicine disease models
Source: Front Toxicol. 2026 Oct 27;8:1951777. doi: 10.3389/ftox.2026.1951777 (PMC13749717; doi:10.3389/ftox.2026.1951777)
Supplement: Supplementary file 6 [file DataSheet6.pdf]

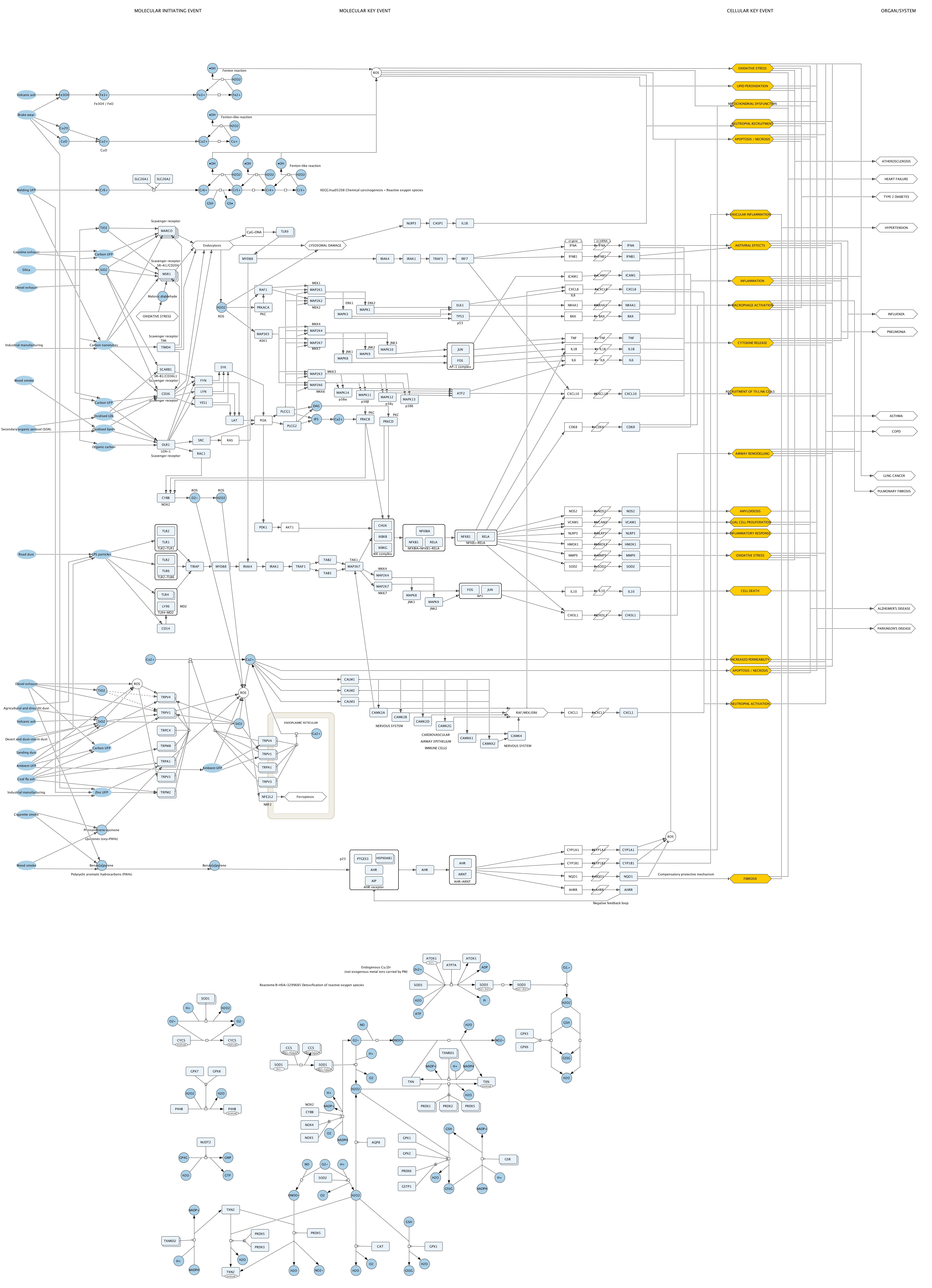

MOLECULAR INITIATING EVENT
MOLECULAR KEY EVENT
CELLULAR KEY EVENT
ORGAN/SYSTEM
Volcanic ash
Brake wear
Welding UFP
Gasoline exhaust
Silica
Diesel exhaust
Industrial manufacturing
Wood smoke
Secondary organic aerosol (SOA)
Road dust
Agricultural and drought dust
Desert and dust-storm dust
Sanding dust
Ambient UFP
Coal fly ash
Cigarette smoke
Fe3O4 / FeO
CuO
Fenton reaction
Fenton-like reaction
KEGG:hsa05208 Chemical carcinogenesis - Reactive oxygen species
SLC26A1
SLC26A2
Scavenger receptor
MARCO
Endocytosis
CpG-DNA
TLR9
LYSOSOMAL DAMAGE
NLRP3
CASP1
IL1B
Scavenger receptor SR-A1/CD204
MSR1
Malonic dialdehyde
OXIDATIVE STRESS
Carbon nanotubes
Scavenger receptor TIM
TIMD4
SCARB1
SR-B1/CD36L1 Scavenger receptor
CD36
Carbon UFP
Oxidised LDL
Oxidised lipids
Organic carbon
OLR1
LOX-1 Scavenger receptor
SYK
FYN
LYN
YES1
LAT
SRC
RAS
RAC1
PI3K
PLCG1
PLCG2
DAG
IP3
Ca2+
PRKCB
PRKCD
PKC
MYD88
IRAK4
IRAK1
TRAF3
IRF7
RAF1
PRKACA
MEK1
MEK2
MAP2K1
MAP2K2
MAPK3
MAPK1
ERK1
ERK2
ELK1
TP53
p53
MAP3K5
ASK1
MKK4
MKK7
MAP2K4
MAP2K7
MAPK8
MAPK9
MAPK10
JNK1
JNK2
JNK3
JUN
FOS
AP-1 complex
MKK3
MKK6
MAP2K3
MAP2K6
MAPK14
MAPK11
MAPK12
MAPK13
p38α
p38β
p38γ
p38δ
ATF2
CYBB
NOX2
O2-
H2O2
ROS
PDK1
AKT1
CHUK
IKBKB
IKBKG
IKK complex
NFKBIA
NFKB1
RELA
NFKBIA-NFKB1-RELA
NFKB1-RELA
LPS particles
TLR2
TLR1
TLR2-TLR1
TLR6
TLR2-TLR6
TLR4
LY96
TLR4-MD2
MD2
CD14
TIRAP
TRAF1
TAB2
TAB3
TAK1
MAP3K7
FOS
JUN
AP1
IFNA
IFNB1
ICAM1
CXCL8
IL8
NR4A1
BAX
TNF
IL1B
IL6
CXCL10
CD68
NOS2
VCAM1
NLRP3
HMOX1
MMP9
SOD2
IL10
CHI3L1
CXCL1
CYP1A1
CYP1B1
NQO1
AHRR
OXIDATIVE STRESS
LIPID PEROXIDATION
MITOCHONDRIAL DYSFUNCTION
NEUTROPHIL RECRUITMENT
APOPTOSIS / NECROSIS
VASCULAR INFLAMMATION
ANTIVIRAL EFFECTS
INFLAMMATION
MACROPHAGE ACTIVATION
CYTOKINE RELEASE
RECRUITMENT OF Th1/NK CELLS
AIRWAY REMODELLING
AMYLOIDOSIS
GLIAL CELL PROLIFERATION
INFLAMMATORY RESPONSE
CELL DEATH
INCREASED PERMEABILITY
NEUTROPHIL ACTIVATION
FIBROSIS
ATHEROSCLEROSIS
HEART FAILURE
TYPE 2 DIABETES
HYPERTENSION
INFLUENZA
PNEUMONIA
ASTHMA
COPD
LUNG CANCER
PULMONARY FIBROSIS
ALZHEIMER'S DISEASE
PARKINSON'S DISEASE
TiO2
SiO2
Zinc UFP
TRPV4
TRPV1
TRPC4
TRPM8
TRPA1
TRPV3
TRPM2
CALM1
CALM2
CALM3
CAMK2A
CAMK2B
CAMK2D
CAMK2G
CAMKK1
CAMKK2
CAMK4
RAF/MEK/ERK
NERVOUS SYSTEM
CARDIOVASCULAR
AIRWAY EPITHELIUM
IMMUNE CELLS
ENDOPLASMIC RETICULUM
NFE2L2
NRF2
Ferroptosis
Phenanthrene-quinone
Quinones (oxy-PAHs)
Benzo[a]pyrene
Polycyclic aromatic hydrocarbons (PAHs)
p23
PTGES3
HSP90AB1
AHR
AIP
AHR receptor
ARNT
AHR-ARNT
Compensatory protective mechanism
Negative feedback loop
Endogenous Cu/Zn
(not exogenous metal ions carried by PM)
Reactome:R-HSA-3299685 Detoxification of reactive oxygen species
ATOX1
ATP7A
Zn2+
SOD3
ADP
ATP
Pi
SOD1
CYCS
GPX7
GPX8
P4HB
NUDT2
GP4G
GMP
GTP
NO
ONOO-
NO2-
CCS
TXNRD1
NADP+
NADPH
H+
TXN
PRDX1
PRDX2
PRDX5
NOX4
NOX5
AQP8
GPX1
GPX2
PRDX6
GSTP1
GSH
GSSG
GSR
GPX5
GPX6
SOD2
TXN2
TXNRD2
PRDX3
CAT
