# Supplementary material for: ExposomeMap-PM: fine and ultrafine particle-induced toxicity - from adverse outcome pathways to systems biomedicine disease models
Source: Front Toxicol. 2026 Oct 27;8:1951777. doi: 10.3389/ftox.2026.1951777 (PMC13749717; doi:10.3389/ftox.2026.1951777)
Supplement: Supplementary file 7 [file DataSheet7.pdf]

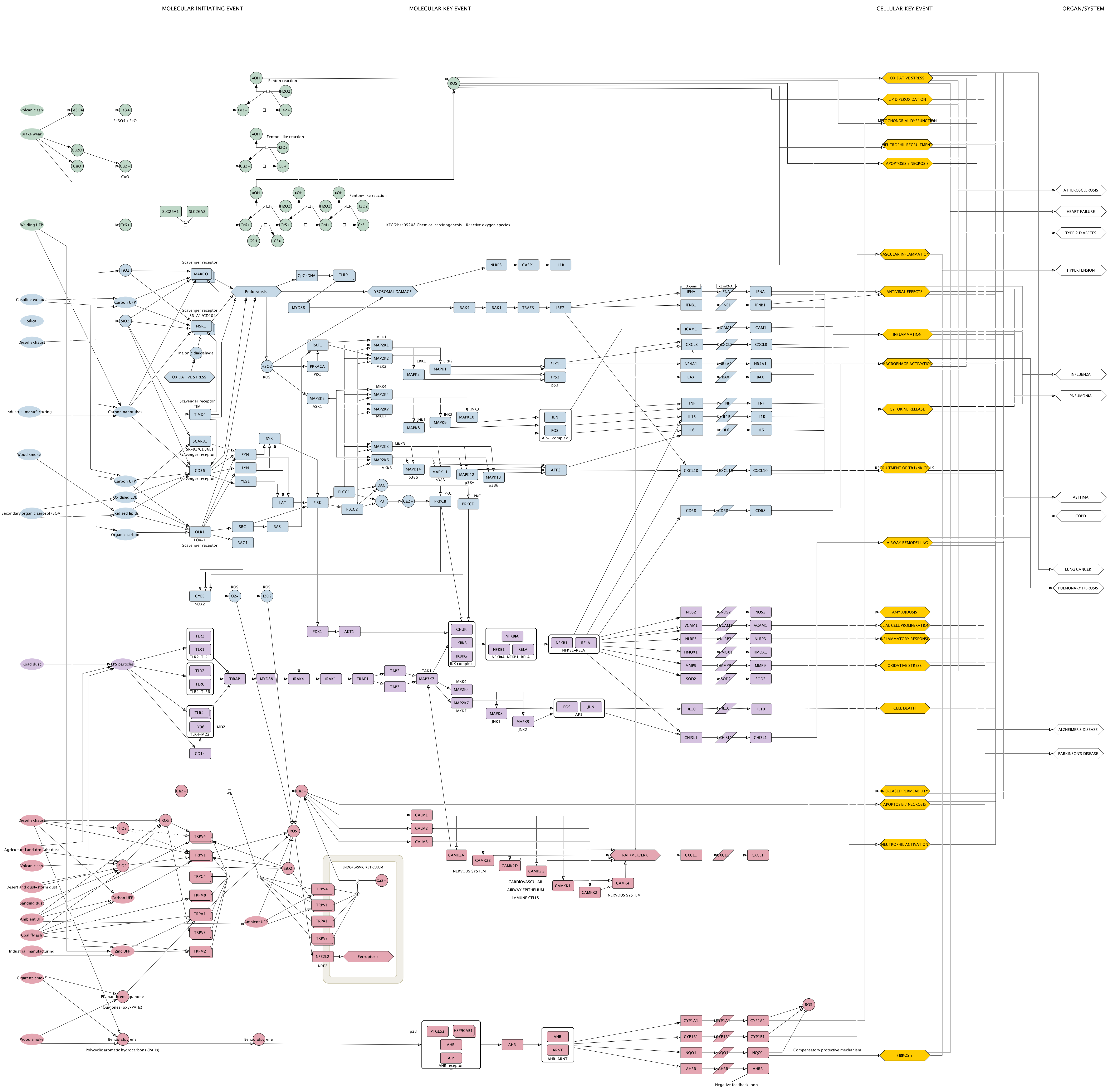
MOLECULAR INITIATING EVENT
MOLECULAR KEY EVENT
CELLULAR KEY EVENT
ORGAN/SYSTEM
Volcanic ash
Brake wear
Welding UFP
Fe3O4
Fe3+
Fe3O4 / FeO
Fe2+
•OH
Fenton reaction
H2O2
Cu2O
CuO
Cu2+
Cu+
Fenton-like reaction
SLC26A1
SLC26A2
Cr6+
Cr5+
Cr4+
Cr3+
GSH
GS•
KEGG:hsa05208 Chemical carcinogenesis - Reactive oxygen species
ROS
Gasoline exhaust
Silica
Diesel exhaust
Industrial manufacturing
Wood smoke
Secondary organic aerosol (SOA)
TiO2
Carbon UFP
SiO2
Carbon nanotubes
Oxidised LDL
Oxidised lipids
Organic carbon
Scavenger receptor
MARCO
Scavenger receptor SR-A1/CD204
MSR1
Malonic dialdehyde
OXIDATIVE STRESS
Scavenger receptor TIM
TIMD4
SCARB1
SR-B1/CD36L1 Scavenger receptor
CD36
Scavenger receptor
OLR1
LOX-1 Scavenger receptor
Endocytosis
CpG-DNA
TLR9
LYSOSOMAL DAMAGE
MYD88
NLRP3
CASP1
IL1B
IRAK4
IRAK1
TRAF3
IRF7
H2O2
RAF1
PRKACA
PKC
MEK1
MAP2K1
MAP2K2
MEK2
ERK1
ERK2
MAPK3
MAPK1
ELK1
TP53
p53
MAP3K5
ASK1
MKK4
MAP2K4
MAP2K7
MKK7
JNK1
JNK2
JNK3
MAPK8
MAPK9
MAPK10
JUN
FOS
AP-1 complex
MAP2K3
MKK3
MAP2K6
MKK6
MAPK14
p38α
MAPK11
p38β
MAPK12
p38γ
MAPK13
p38δ
ATF2
SYK
FYN
LYN
YES1
LAT
PI3K
PLCG1
PLCG2
DAG
IP3
Ca2+
PRKCB
PRKCD
SRC
RAS
RAC1
CYBB
NOX2
O2−
IFNA
IFNB1
ICAM1
CXCL8
NR4A1
BAX
TNF
IL1B
IL6
CXCL10
CD68
OXIDATIVE STRESS
LIPID PEROXIDATION
MITOCHONDRIAL DYSFUNCTION
NEUTROPHIL RECRUITMENT
APOPTOSIS / NECROSIS
VASCULAR INFLAMMATION
ANTIVIRAL EFFECTS
INFLAMMATION
MACROPHAGE ACTIVATION
CYTOKINE RELEASE
RECRUITMENT OF Th1/NK CELLS
AIRWAY REMODELLING
ATHEROSCLEROSIS
HEART FAILURE
TYPE 2 DIABETES
HYPERTENSION
INFLUENZA
PNEUMONIA
ASTHMA
COPD
LUNG CANCER
PULMONARY FIBROSIS
Road dust
LPS particles
TLR2
TLR1
TLR2-TLR1
TLR6
TLR2-TLR6
TLR4
LY96
TLR4-MD2
MD2
CD14
TIRAP
TRAF1
TAB2
TAB3
TAK1
MAP3K7
PDK1
AKT1
CHUK
IKBKB
IKBKG
IKK complex
NFKBIA
NFKB1
RELA
NFKBIA-NFKB1-RELA
NFKB1-RELA
AP1
NOS2
VCAM1
HMOX1
MMP9
SOD2
IL10
CHI3L1
AMYLOIDOSIS
DUAL CELL PROLIFERATION
INFLAMMATORY RESPONSE
OXIDATIVE STRESS
CELL DEATH
ALZHEIMER'S DISEASE
PARKINSON'S DISEASE
Diesel exhaust
Agricultural and drought dust
Volcanic ash
Desert and dust-storm dust
Sanding dust
Ambient UFP
Coal fly ash
Industrial manufacturing
Zinc UFP
TRPV4
TRPV1
TRPC4
TRPM8
TRPA1
TRPV3
TRPM2
ENDOPLASMIC RETICULUM
NFE2L2
NRF2
Ferroptosis
CALM1
CALM2
CALM3
CAMK2A
CAMK2B
CAMK2D
CAMK2G
CAMKK1
CAMKK2
CAMK4
RAF/MEK/ERK
CXCL1
NERVOUS SYSTEM
CARDIOVASCULAR
AIRWAY EPITHELIUM
IMMUNE CELLS
INCREASED PERMEABILITY
APOPTOSIS / NECROSIS
NEUTROPHIL ACTIVATION
Cigarette smoke
Phenanthrene-quinone
Quinones (oxy-PAHs)
Wood smoke
Benzo[a]pyrene
Polycyclic aromatic hydrocarbons (PAHs)
p23
PTGES3
HSP90AB1
AHR
AIP
AHR receptor
ARNT
AHR-ARNT
CYP1A1
CYP1B1
NQO1
AHRR
Compensatory protective mechanism
FIBROSIS
Negative feedback loop

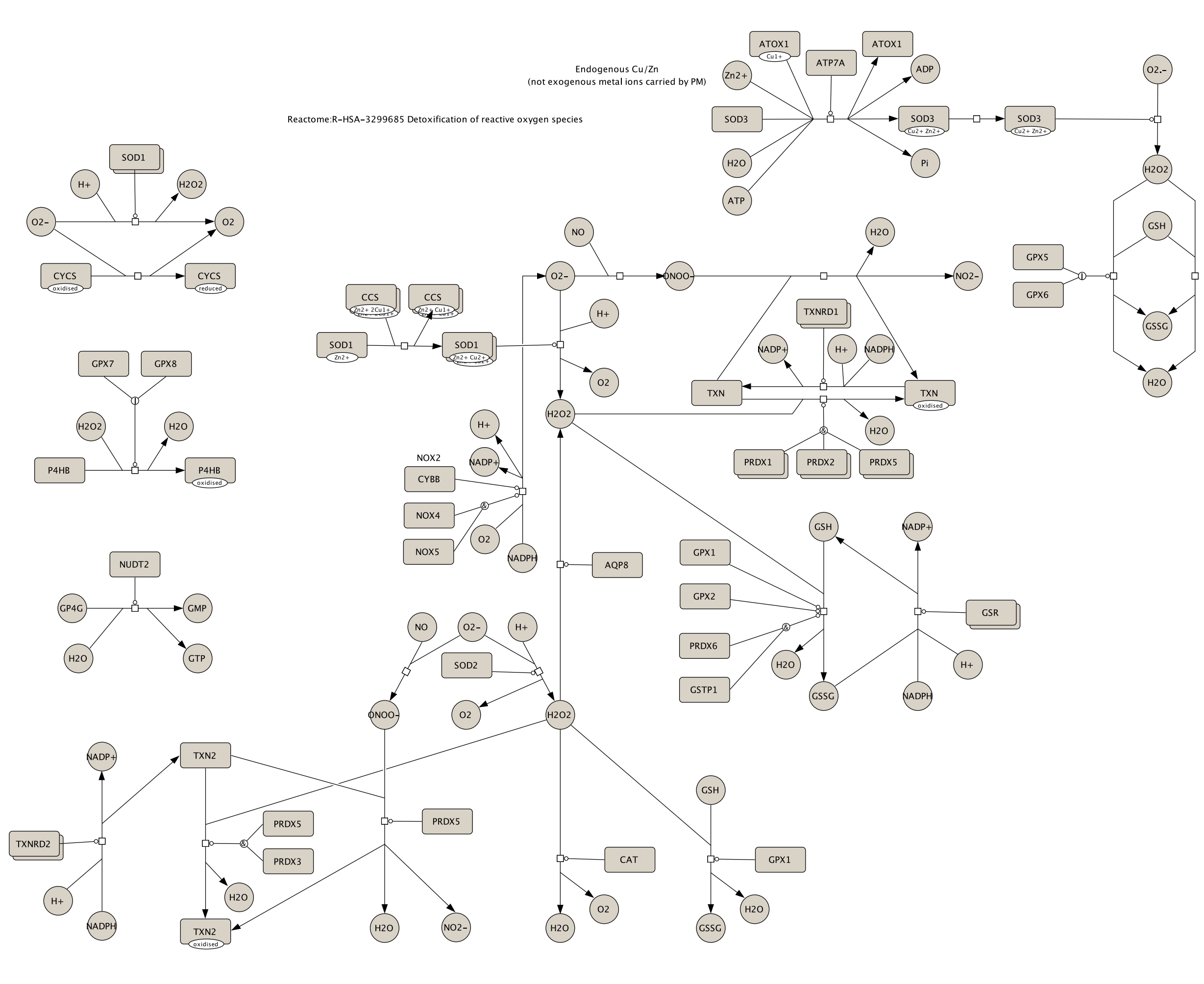
Endogenous Cu/Zn
(not exogenous metal ions carried by PM)
Reactome:R-HSA-3299685 Detoxification of reactive oxygen species
ATOX1
ATP7A
Zn2+
SOD3
ADP
H2O
ATP
Pi
O2−
H2O2
GSH
GPX5
GPX6
GSSG
SOD1
H+
O2
CYCS
GPX7
GPX8
P4HB
NUDT2
GP4G
GMP
GTP
NO
ONOO−
NO2−
CCS
TXNRD1
NADP+
NADPH
TXN
PRDX1
PRDX2
PRDX5
NOX2
CYBB
NOX4
NOX5
AQP8
GPX1
GPX2
PRDX6
GSTP1
GSR
SOD2
TXN2
TXNRD2
PRDX3
CAT
